# FaRIF as a key regulator of strawberry fruit ripening: deciphering its targets and interaction networks

**DOI:** 10.1093/hr/uhaf362

**Published:** 2026-01-06

**Authors:** Carmen Martín-Pizarro, María Florencia Perotti, José M Franco-Zorrilla, Rosa Lozano-Durán, Guozheng Qin, David Posé

**Affiliations:** Instituto de Hortofruticultura Subtropical y Mediterránea (IHSM) “La Mayora,” Universidad de Málaga-Consejo Superior de Investigaciones Científicas, Laboratorio de Bioquímica y Biotecnología Vegetal, Departamento de Biología Molecular y Bioquímica, Facultad de Ciencias, UMA, 29010 Málaga, Spain; Unidad Asociada de I+D+i IFAPA-CSIC Biotecnología y Mejora en Fresa, Málaga, Spain; Instituto de Hortofruticultura Subtropical y Mediterránea (IHSM) “La Mayora,” Universidad de Málaga-Consejo Superior de Investigaciones Científicas, Laboratorio de Bioquímica y Biotecnología Vegetal, Departamento de Biología Molecular y Bioquímica, Facultad de Ciencias, UMA, 29010 Málaga, Spain; Unidad Asociada de I+D+i IFAPA-CSIC Biotecnología y Mejora en Fresa, Málaga, Spain; Department of Plant Molecular Genetics, Centro Nacional de Biotecnología, CNB-CSIC, 28049 Madrid, Spain; Department of Plant Biochemistry, Center for Plant Molecular Biology (ZMBP), Eberhard Karls University, 72076 Tübingen, Germany; State Key Laboratory of Plant Diversity and Specialty Crops, Institute of Botany, Chinese Academy of Sciences, Beijing 100093, China; Instituto de Hortofruticultura Subtropical y Mediterránea (IHSM) “La Mayora,” Universidad de Málaga-Consejo Superior de Investigaciones Científicas, Laboratorio de Bioquímica y Biotecnología Vegetal, Departamento de Biología Molecular y Bioquímica, Facultad de Ciencias, UMA, 29010 Málaga, Spain; Unidad Asociada de I+D+i IFAPA-CSIC Biotecnología y Mejora en Fresa, Málaga, Spain

## Abstract

Ripening inducing factor (RIF) is a key NAC transcription factor (TF) regulating strawberry fruit ripening. Previous studies using *RIF-*RNAi and overexpression lines in *Fragaria × ananassa* and CRISPR knock-out lines in *F. vesca* have established the role of RIF in controlling ABA biosynthesis and signaling, cell wall remodeling, and secondary metabolism. In this study, we deciphered FaRIF’s transcriptional regulatory network by combining ChIP-seq-based identification of its direct targets with an analysis of *FaRIF*-RNAi transcriptome data. These analyses revealed FaRIF’s direct role in multiple aspects of strawberry fruit ripening, including the regulation of ripening-related TFs, phytohormone content and signaling, primary and secondary metabolism, and cell wall degradation. Additionally, using the TurboID-based proximity labeling approach, we have identified FaRIF interactors, including proteins involved in mRNA and protein homeostasis, as well as several NAC TFs. Among these, FaNAC021 and FaNAC034 were found to potentially cooperate with FaRIF to enhance the transcription of shared target genes. This integrative analysis, combining transcriptome analysis, *in vivo* ChIP-seq, and proximity labeling, broadens our understanding of FaRIF-mediated transcriptional networks and interaction partners, providing valuable insights into the molecular regulation of strawberry fruit ripening by this TF.

## Introduction

Cultivated strawberry (*Fragaria × ananassa* Duch.) is one of the most economically significant berry crops worldwide [1], primarily due to its unique organoleptic attributes and nutritional value [2–4]. These characteristics develop during fruit ripening, a highly coordinated developmental process that has evolved to facilitate seed dispersal, an essential factor in evolutionary success [5]. Ripening is characterized by the softening of the fruit and an increase in the sugars, flavor-enhancing volatiles, and essential micronutrient content. The mechanisms underlying these processes primarily rely on the action of phytohormones [6], epigenetic modifications [7, 8], and transcription factors (TFs) [9].

As a nonclimacteric fruit, strawberry ripening is mainly regulated by abscisic acid (ABA) [10–12], whose levels increase during fruit ripening [13–15]. In addition to ABA, other phytohormones also contribute to strawberry fruit development and ripening [6]. Notably, auxins and gibberellins play pivotal roles during early developmental stages, inhibiting ripening by suppressing ABA accumulation through the activation of *CYP707A4a* expression, a cytochrome P450 monooxygenase involved in ABA catabolism [16]. Epigenetic modifications, including changes in DNA methylation [17–19] and histone modifications [8], have also been reported to influence strawberry ripening, further underscoring their significance in this process.

In recent years, a number of TFs have been identified as important regulators of ripening, most of which have a specific role in a particular ripening-related process [9]. Among these, MYB10, a member of the R2R3-type MYB TF family, is a key regulator of anthocyanin biosynthesis and probably the most extensively studied TF in strawberry [20–26]. However, despite significant progress in understanding the role of TFs in strawberry fruit ripening regulation, the transcriptional regulatory networks they control and the mechanisms regulating their activity still remain poorly characterized.

NAC (NAM, ATAF, and CUC) TFs constitute a large plant-specific family involved in diverse developmental processes and responses to environmental stimuli [27], with a number of them being involved in fruit ripening in different species [28, 29]. These proteins are characterized by a conserved N-terminal region, known as the NAC domain, responsible for DNA recognition, binding, and dimerization, and a variable C-terminal region that defines the different NACs subgroups. In the woodland strawberry *F. vesca*, one of the diploid ancestor species of *F. × ananassa* [30], 112 *NAC* genes have been identified [31], although only two have been studied in detail. One of these, *FcNAC1*, the *F. chiloensis* ortholog of *NAC022*, was shown to activate the expression of *FcPL*, a gene encoding the cell wall remodeling enzyme pectate lyase [32]. Recently, RNAi-silenced and overexpression lines of this gene in *F. vesc*a (renamed *FvNAC073* based on its closest Arabidopsis ortholog) also supported the role of this TF in cell wall remodeling, as well as provided additional functions, including regulation of ABA and anthocyanin biosynthesis, and sucrose accumulation [33, 34].

The second functionally characterized NAC TF in strawberry is *NAC035*, also known as *Ripening Inducing Factor* (*RIF*) in both *F. × ananassa* (*FaRIF*) and *F. vesca* (*FvRIF*) [35, 36]. In these studies, stable RNAi-silencing and CRISPR knockout lines were established in each species, along with overexpression lines in both. Transcriptomic and metabolomic profiling, combined with phenotypic characterization in *F. × ananassa* lines with altered *FaRIF* expression, revealed that this TF regulates several ripening-associated processes, including cell wall degradation, anthocyanin biosynthesis, and the accumulation of sugars, organic acids, and volatiles. It also influences the aerobic/anaerobic metabolic balance, a key determinant in the onset of strawberry fruit ripening [37]. Notably, FaRIF was shown to regulate ABA accumulation, suggesting an upstream regulatory role for this TF [36]. The characterization of its ortholog, *FvRIF*, in *F. vesca* further confirmed its central regulatory role of this TF, displaying the *Fvrif* knockout mutant lines a complete blockage of the ripening process [35]. In this study, FvRIF was shown to interact with and serves as a substrate for MAP kinase 6 (FvMAPK6), with the phosphorylation at Thr-310 being essential for its transcriptional activity. Additionally, the DNA binding sites of FvRIF using DNA affinity purification sequencing (DAP-seq) were identified, revealing several structural genes involved in the anthocyanin pathway, cell wall degradation, sugar metabolism, and aroma compounds biosynthesis among its direct target genes.

DAP-seq is a powerful, fast, and cost-effective technique for identifying DNA binding sites of TFs or other DNA-associated proteins [38]. To date, and in addition to the study of FvRIF, only a few recent studies have employed this approach to identify TF target genes in strawberry, specifically in *F. vesca*. These include the APETALA2 (AP2) TF BARE RECEPTACLE (BRE) [39], involved in floral organogenesis, as well as FvTCP7 [40] and FvSEP3 [41], both implicated in the regulation of fruit ripening. However, although of great value, DAP-seq, as an *in vitro* approach, lacks the chromatin context of *in vivo* interactions and does not account for the influence of other protein interactors or cofactors on TF binding sites, which can be captured using Chromatin Immunoprecipitation (ChIP) followed by deep sequencing (ChIP-seq). Probably due to the extremely recalcitrant nature of strawberry, ChIP has only been applied, and only followed by qPCR validation, in a few studies in strawberry to validate target genes for FvWRKY48 [42], BRE [3, 9], and FvRIF itself [35]. Notably, only a few examples of ChIP-seq application have been reported in this species, focusing on the study of chromatin states through histone modification analysis [8, 43–45], while no studies to date have addressed the identification of TF target genes.

In this work, we have advanced our understanding of FaRIF role in the regulation of strawberry fruit ripening. First, we have set up a protocol for successfully performing a ChIP-seq experiment on a TF and we have identified its target genes *in vivo* in *F. × ananassa.* By integrating these results with the analysis of a transcriptome dataset from *RIF*-RNAi lines, mapped to the octoploid reference genome annotation of *F. × ananassa* cv. Camarosa [46], we uncovered the gene regulatory network governed by this TF, both directly and indirectly.

Additionally, we optimized and applied the TurboID-based proximity labeling approach in strawberry fruits to investigate FaRIF’s proximal interactome *in vivo.* This analysis revealed a diverse set of putative FaRIF interactors, including other NAC family members, such as the ripening-related FaNAC021 and FaNAC034, as well as proteins involved in processes like protein folding and mRNA stability. In summary, this study provides valuable insights into the regulation underpinning the complex ripening process in strawberry mediated by this key TF. Furthermore, it offers detailed methodologies that will benefit researchers interested in applying these omics approaches to a complex species like strawberry.

## Results

### Analysis of FaRIF protein homoeologs and characterization of their expression profile in *F.* × ananassa cv. Camarosa

**Figure 1:**
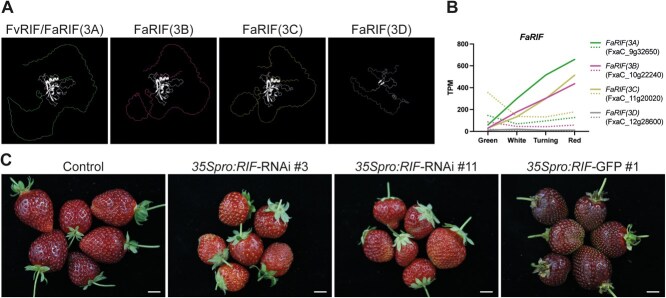
Protein structures, gene expression during strawberry fruit ripening, and phenotypes of *FaRIF* silencing and overexpression lines*.* (**A**) Predicted protein structures of FvRIF and FaRIFs homoeolog proteins using Alphafold 3 [48]. The predicted models were aligned to model 0 of the FvRIF/FaRIF(3A) protein using PyMol. The NAC domain is shown in white. (**B**) Expression patterns of the four *FaRIF* homoeologs in *F.* × *ananassa* at four ripening stages in receptacles (continuous lines) and achenes (dotted lines). Data from [46, 49]. Colors denote the four subgenomes encoding each *FaRIF* homoeolog: green (*F. vesca*), purple (*F. iinumae*), yellow (*F. nipponica*), and grey (*F. viridis*). (**C**) Fruit phenotype at the red stage in control and stable transgenic lines *35Spro:RIF-*RNAi and *35Spro:RIF-GFP*, which show significant downregulation and upregulation of *FaRIF*, respectively [36]. Scale bars = 1 cm.

*FaRIF*, whose ortholog in *F. vesca* (*FvRIF*) is encoded by the gene FvH4_3g20700, is encoded by four homoeologs in the *F.* × ananassa cv. Camarosa genome [30, 46], located on chromosomes 3A (*FaRIF(3A)*, FxaC_9g32650, or maker-Fvb3-4-augustus-gene-182.31), 3B (*FaRIF(3B)*, FxaC_10g22240, or augustus_masked-Fvb3-2-processed-gene-121.1), 3C (*FaRIF(3C)*, FxaC_11g20020, or maker-Fvb3-3-augustus-gene-106.29) and 3D (*FaRIF(3D)*, FxaC_12g28600, or maker-Fvb31-augustus-gene-197.26; Dataset S1), according to the chromosome nomenclature suggested by Hardigan and collaborators [47]. Protein sequence alignment of the four homoeologs revealed that FaRIF(3A), (3B), and (3C) shared most residues with FvRIF, with FaRIF(3A) exhibiting an identical protein sequence to the latter (Fig. S1). However, *FaRIF(3D)* encoded for a shorter protein, lacking the first 144 residues of the conserved N-terminal region and consequently missing most of the NAC domain (Fig. S1). A protein structure prediction using AlphaFold 3 [48] (Figs 1A and S2) indicated the absence in FaRIF(3D) of essential residues needed for DNA binding and NAC protein dimerization [50], suggesting that this homoeolog is nonfunctional.

Based on the reanalysis of the transcriptome changes during *F.* × ananassa cv. Camarosa ripening [46], and as previously reported for *FvRIF* [35] and for *FaRIF* using the *F. vesca* as the reference genome [36], *FaRIF* expression increased during the ripening process of receptacles (Fig. 1B). This expression pattern was observed for all *FaRIF* homoeologs, except for the truncated homoeolog *FaRIF(3D)*, whose expression remained low throughout the process, supporting that this subgenome copy does not contribute to ripening regulation. Notably, *FaRIF(3A)*, the identical orthologue to *FvRIF* located in the *F. vesc*a’s subgenome, was the most highly expressed *FaRIF* homoeolog in receptacles. By contrast, *FaRIF* expression in achenes followed a different pattern: with the exception of *FaRIF(3D)*, which was also minimally expressed in this tissue, all other homoeologs showed a decrease from the green to the white stage, followed by a slight increase toward the ripe stage (Fig. 1B). Among them, *FaRIF(3C)* was the most expressed homoeolog in achenes. Remarkably, the expression levels of all full-length *FaRIF* homoeologs were significantly higher in receptacles than in achenes.

### Identification of genome-wide *in vivo* FaRIF binding sites

**Figure 2:**
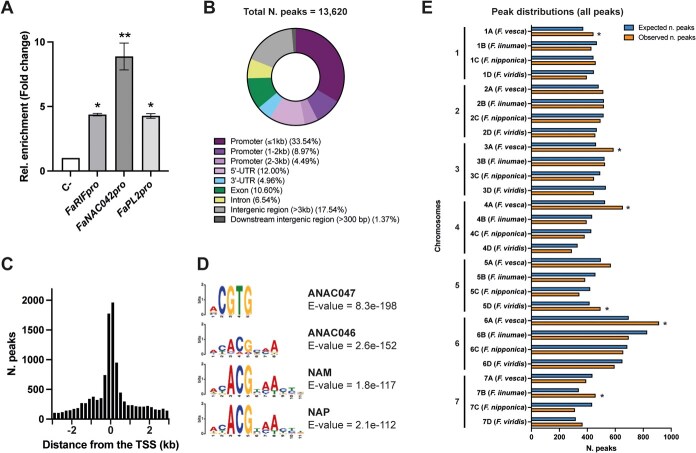
Genome-wide identification of FaRIF binding sites through ChIP-seq. (**A**) ChIP-qPCR assay showing the enrichment (Fold Change (FC)) of binding of FaRIF-GFP to the promoters of *FaRIF*, *FaNAC042*, and *FaPL2/plB*, relative to the negative control (FxaC_21g51230). Statistically significant enrichment is indicated by asterisks (^**^P ≤0.005; ^*^P ≤ 0.05). (**B**) Distribution of FaRIF binding sites across genome features. (**C**) Distribution of FaRIF binding sites 3 kb upstream and downstream of the TSS. (**D**) Most enriched NAC DNA logos for FaRIF DNA binding sites, corresponding to the mentioned Arabidopsis genes. (**E**) Number of observed (orange bars) and expected (blue bars) binding sites for all peaks consistently identified in both biological replicates. The expected number of binding sites was calculated by dividing the total number of peaks for each chromosome set by the total size of that set. Asterisks (*) indicate statistically significant differences between observed and expected binding sites.

Using our previously established GFP-tagged *FaRIF* overexpression line in *F.* × ananassa cv. Camarosa (*35Spro:RIF-GFP* #1), which overexpresses the *FaRIF(3B)* homoeolog (Fig. 1C) [36], we aimed to elucidate the *in vivo* binding sites of FaRIF through a ChIP-seq assay (Table S1). To validate the ChIP assay, we analyzed the enrichment of two known direct targets of FvRIF (*F. vesca*’s ortholog) [35]: *NAC042,* a ripening-induced NAC TF [36], and *PECTATE LYASE 2* (*PL2/plB*), involved in cell wall disassembly [51]*,* along with *FaRIF* itself. As a negative control, we selected a locus not expected to bind FaRIF (the first exon of FxaC_21g51230, a gene not expressed in fruits [46])*.* As shown in Fig. 2A, the two biological replicates of the FaRIF-GFP samples exhibited at least 4-fold enrichment for the three expected target genes, in contrast to the negative control. This validated the ChIP assay and supported the binding of both FvRIF and FaRIF to the promoters of *NAC042* and *PL2/plB*, as well as to its own promoter, suggesting that FaRIF may control its own expression through feedback regulation.

Subsequently, we performed high-throughput sequencing of the ChIP samples. A total of 13 620 peaks were identified across the two biological replicates analyzed and were therefore considered high-confidence binding sites for FaRIF (Table S1). A peak distribution analysis showed that most binding sites were located within promoter regions, with 59% positioned within 3 kb upstream of the transcription start site (TSS), primarily 1 kb upstream (Fig. 2B). Additionally, examining a 3-kb region surrounding the TSS revealed that most peaks were centered on the TSS (Fig. 2C). A MEME analysis further identified significant enrichment of known NAC recognition motifs among the FaRIF binding sites, including those corresponding to the Arabidopsis NACs ANAC047, ANAC046, NAM (the closest homolog to FaRIF), and NAP (Fig. 2D), supporting the effectiveness of the ChIP-seq experiment. Nevertheless, to provide additional layers of evidence supporting target gene assignment, we applied further stringent criteria to filter the ChIP-seq peaks and defined corresponding validation levels (VLs) for each: (1) total peaks found in both biological replicates with a fold-enrichment >2 and a q-value <0.001 (VL1); (2) those in VL1 containing peaks with reproducible summits within ≤200 bp and an irreproducible discovery rate (IDR) ≤0.05 (VL2); and (3) those in VL1 containing peaks with reproducible summits within ≤100 bp and an IDR ≤0.05 (VL3; Tables S2, S5, S6, and  S7).

Next, we examined whether FaRIF binding sites exhibit positional bias in the genome by analyzing peak distribution across the homoeologous chromosomes of the four parental subgenomes of *F.* × *ananassa*, within the seven sets of chromosomes. While no preference was observed for chromosome 2, all other chromosomes exhibited higher representation of FaRIF binding sites in specific subgenomes (Fig. 2E and Table S2). Notably, the *F. vesca* subgenomes contained more FaRIF binding sites on chromosomes 1, 3, 4, and 6 compared to the other subgenomes. Focusing on peaks located 2 kb upstream to 100 bp downstream of the TSS, regions most likely influencing gene expression regulation, we observed a similar FaRIF preference, except for chromosome 1, which showed no bias (Fig. S3 and Table S2). These findings are consistent with the reported subgenome dominance of *F. vesca* in *F.* × *ananassa* [30].

### Identification of genes directly and indirectly regulated by FaRIF

**Figure 3:**
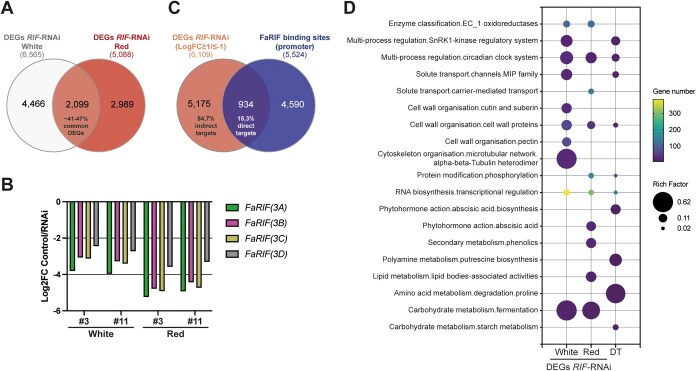
RNA-seq and ChIP-seq data reveal biological processes directly and indirectly regulated by FaRIF. (**A**) Comparison of DEGs in receptacles of both independent *FaRIF-*RNAi lines (*35Spro:RIF-*RNAi #3 and #11) at the white and red ripening stages. (**B**) Expression of the four *FaRIF* homoeologs (Log2(FC) control/RNAi ratio) in receptacles of both independent *FaRIF-*RNAi lines at the white and red ripening stages. (**C**) Comparison of all DEGs identified in *FaRIF*-RNAi lines at both ripening stages and genes with FaRIF binding sites in the promoter regions (VL1). (**D**) MapMan enrichment analysis of DEGs in *FaRIF-*RNAi receptacles (≥2-fold upregulation or downregulation and FDR ≤ 0.05 in the RNAi lines) at white and red stages and of FaRIF direct targets (DT) at VL1. Top ten most statistically significant MapMan second-level bins levels for each analysis were selected for representation. Unique lower-level bins found within the top 10 were also included.

To identify genes transcriptionally regulated by FaRIF, we reanalyzed previously generated RNA-seq data from receptacles at white and red stages of control and two RNAi lines (*35Spro:RIF-*RNAi #3 and #11; Fig. 1C). These datasets, initially mapped to the *F. vesca* reference genome [36], were re-mapped here to the *F.* × *ananassa* cv. Camarosa genome assembly v1.0.a1 [30] using the v1.0.a2 annotation version [46]. With a mapping rate ranging from a minimum of 88.7% to a maximum of 97.5%, of the 108 447 annotated genes in the *F.* × *ananassa* genome, 6565 and 5088 genes were found differentially expressed (DEGs; false discovery rate (FDR) *P-value* correction ≤0.05) in both *FaRIF*-RNAi lines at the white and red stages, respectively, with 2099 shared between the two RNAi lines (Fig. 3A, Table S3). Furthermore, approximately 50% of the DEGs were either up- or downregulated at each ripening stage (Table S3). Confirming the efficiency of the RNAi-mediated silencing, transcript levels of the four *FaRIF* homoeologs were significantly reduced in both transgenic lines and at both ripening stages (Fig. 3B, Table S3).

To distinguish between direct and indirect FaRIF targets, we selected DEGs with at least a two-fold change (downregulated or upregulated; Log_2_(FC) ≤ −1 or ≥ 1; FDR ≤ 0.05) across both stages in *FaRIF-*RNAi receptacles (6109 genes), and overlapped these with genes containing FaRIF binding sites within their promoter regions (from 2 kb upstream to 100 bp downstream of the TSSs). At VL1, 5524 genes contained FaRIF binding sites within this selected promoter region. This number was reduced to 4126 and 3045 genes at VL2 and VL3, respectively (Table S1 and Fig. S4). Comparison of these datasets revealed that from 15.3% at VL1 (934 *F.* × *ananassa* genes corresponding to 718 unique *F. vesca* homoeologs) to 8.3% at VL3 (504 *F.* × *ananassa* genes corresponding to 416 unique *F. vesca* homoeologs) of the DEGs were direct FaRIF targets (Fig. 3C; Fig. S4A, and Table S4). Among these, from 581 (VL1) to 323 (VL3) genes (61.2% and 63%, respectively) were downregulated, while from 369 (VL1) to 190 (VL3; 38.8% and 37%, respectively) were upregulated (Table S4), suggesting that FaRIF primarily functions as a transcriptional activator. The remaining 84.7% of DEGs (5175 *F.* × *ananassa* genes, corresponding to 3484 unique *F. vesca* homoeologs) did not contain FaRIF binding sites within their promoters and were thus considered indirect targets.

We next conducted a MapMan analysis [52] to identify enriched functional categories among DEGs with more than a two-fold change in expression in the *FaRIF*-RNAi lines at both ripening stages (Fig. 3D, Table S5). This analysis revealed enrichment in processes such as transcriptional regulation, enzyme activity, cell wall organization, and fermentation. At the red stage specifically, categories related to ABA and phenolic compounds were also significantly enriched, supporting the role of FaRIF in directly or indirectly controlling key ripening-related processes. We then performed this analysis on the FaRIF direct targets identified at VL1, VL2, and VL3 (Fig. 3D, Fig. S4B, and Table S5). Notably, the enriched functional categories were broadly consistent across the three VLs, reinforcing the robustness of the ChIP-seq dataset. Interestingly, this analysis further supported that FaRIF directly regulates genes involved in transcriptional regulation, cell wall organization, and ABA biosynthesis, among others, highlighting its essential role in directly modulating these ripening-related processes.

### FaRIF regulates transcriptional cascades involved in strawberry ripening regulation

**Figure 4:**
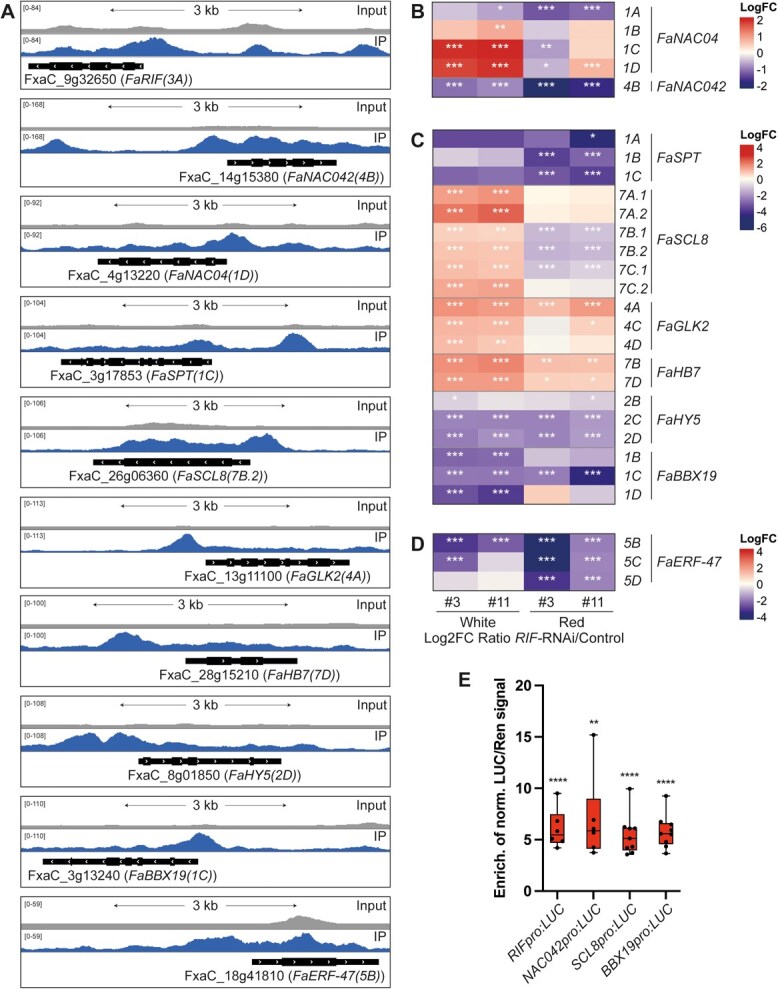
TFs directly regulated by FaRIF. (**A**) ChIP-seq peaks showing FaRIF binding sites and input reads over representative homoeologs of ripening-related TFs. (**B**) to (**D**) Heatmaps displaying the expression of FaRIF-regulated TFs. Panels include TFs from the NAC family (**B**), involved in fruit development and ripening-related processes (**C**), and aroma compounds biosynthesis (**D**). Significant DEGs are marked with asterisks (^***^FDR ≤0.0005; ^**^FDR ≤ 0.005; ^*^FDR ≤ 0.05). Detailed information is available in Table S6. (**E**) Activation of *FaRIF*, *FaNAC042*, *FaSCL8*, and *FaBBX19* promoters by FaRIF in a dual-luciferase reporter assay in infiltrated *N. benthamiana* leaves. Firefly LUCIFERASE (LUC) activity is presented relative to RENILLA (REN) activity and normalized against the control (empty effector vector). Each value represents one biological replicate, each with 3 technical replicates. Statistically significant activation of target genes is indicated by asterisks (^****^P ≤ 0.0001; ^**^P ≤ 0.01).

Within the MapMan bin related to transcriptional regulation, several TFs previously implicated in fruit development and/or ripening were identified among the DEGs (Table S6). First, *FaRIF*—specifically the most expressed homoeolog, *FaRIF(3A)*—was found among the direct targets (Fig. 4A), as previously confirmed by ChIP-qPCR, suggesting a positive feedback regulation of its own expression. Homoeologs of five additional ripening-induced NACs (*FaNAC04*, *FaNAC06*, *FaNAC022*, *FaNAC033*, and *FaNAC042*), were also differentially expressed in at least one ripening stage (Figs 4B and S6A), with homoeologs of *FaNAC04* and *FaNAC042* additionally identified as FaRIF-direct targets (Fig. 4A and B).

Other TFs previously reported to regulate strawberry fruit development and ripening were identified as FaRIF direct targets (Fig. 4C, Fig. S6B, and Table S6). These included all homoeologs of the bHLH TF *SPATULA* (*FaSPT*), involved in strawberry fruit development [53] and sugar accumulation during ripening [40], with three homoeologs downregulated in the *FaRIF*-RNAi lines. Similarly, all homoeologs of *SCARECROW-LIKE8* (*FaSCL8*), encoding a GRAS protein that regulates anthocyanin, soluble sugar accumulation, and fruit firmness [54, 55], were identified as direct targets, with some downregulated at the red stage. All homoeologs of the MYB TF *GOLDEN2-LIKE* (*FaGLK2*)*,* also known as *FvMYBR139* in *F. vesca* [56], were also identified as direct targets and upregulated in the RNAi lines, suggesting that FaRIF may directly and positively regulate strawberry degreening during ripening, as the tomato orthologue of this TF promotes plastid formation and high chlorophyll levels [57]. Additionally, all homoeologs of the homeodomain-leucine zipper (HD-Zip) class I TF *FaHB7*, whose Arabidopsis orthologue, *AtHB7*, acts as a negative regulator of ABA signaling [58], were significantly upregulated at both stages, with two identified as direct targets. Furthermore, most homoeologs of the bZIP *ELONGATED HYPOCOTYL5* (*FaHY5*) and the B-box gene *FaBBX19*, both associated with ABA signaling in Arabidopsis [59, 60], were downregulated at both stages, with three identified as direct FaRIF targets. These findings further support FaRIF’s role in modulating ABA signaling, contributing to the delayed ripening phenotype observed in the *FaRIF-*RNAi lines (Fig. 1C) [36].

In addition to the direct targets, several important TFs involved in the biosynthesis of anthocyanins and/or proanthocyanidins (PA) were found indirectly regulated by FaRIF (Fig. S6C, Table S6). These included homoeologs of the MADS-box TF *SHATTERPROOF-like* (*FaSHP*) [61], the MYB TFs *FaMYB52* [62], *FaMYB77* [63], *FaMYB10(1B)*, the dominant homoeolog of the key regulator of anthocyanin biosynthesis [20], and *FaMYB123* (*F.* vesca’s *FvMYB79* [64]) [64, 65]. Additionally, homoeologs of the B-box gene *FaBBX22*, known to cooperate with *FaHY5* in light-induced anthocyanin biosynthesis [66], and of *PACLOBUTRAZOL RESISTANCE1* (*FaPRE1*), an atypical HLH TF promoting anthocyanin biosynthesis in leaves when stably overexpressed [67], were also differentially expressed. Together, these results support that the altered flavonoid, and particularly anthocyanin, content in the *FaRIF*-RNAi lines might be explained by the broad disturbance in the expression of all these regulators.

Furthermore, TFs involved in volatile organic compounds (VOCs) biosynthesis were also found among the direct and indirect targets of FaRIF (Figs 4D, S6D, and Table S6). Thus, homoeologs of *FaDOF2* and *EMISSION OF BENZENOID II* (*FaEOBII*), encoding DOF-type and MYB TFs, respectively, which synergistically enhance eugenol biosynthesis [68, 69], were indirectly downregulated at the red stage. Furthermore, homoeologs of *FaERF-47*, an AP2/ERF TF linked to volatile esters biosynthesis through activation of the acyltransferase-encoding gene *AAT* in *F. vesca* [70], were identified as direct targets of FaRIF and significantly downregulated at both ripening stages.

To confirm the direct regulatory effect of FaRIF on some of these TFs, we performed transactivation assays using the promoters of *FaRIF* itself, *FaNAC042*, the ripening-associated *FaSCL8*, and the ABA-related *FaBBX19*, each fused to the Firefly *LUCIFERASE* (*LUC*)*.* As shown in Fig. 4E, coexpression in *Nicotiana benthamiana* leaves with FaRIF resulted in increased transcription compared to the negative control (empty vector), confirming FaRIF-mediated activation of all four promoters and supporting their status as biologically relevant direct targets.

In summary, these results highlight a complex regulation of the strawberry fruit development and ripening processes, with FaRIF emerging as a more prominent upstream direct and indirect regulator of several important ripening-related TFs than previously reported in our earlier work [36].

### FaRIF directly regulates cell wall remodeling genes

**Figure 5:**
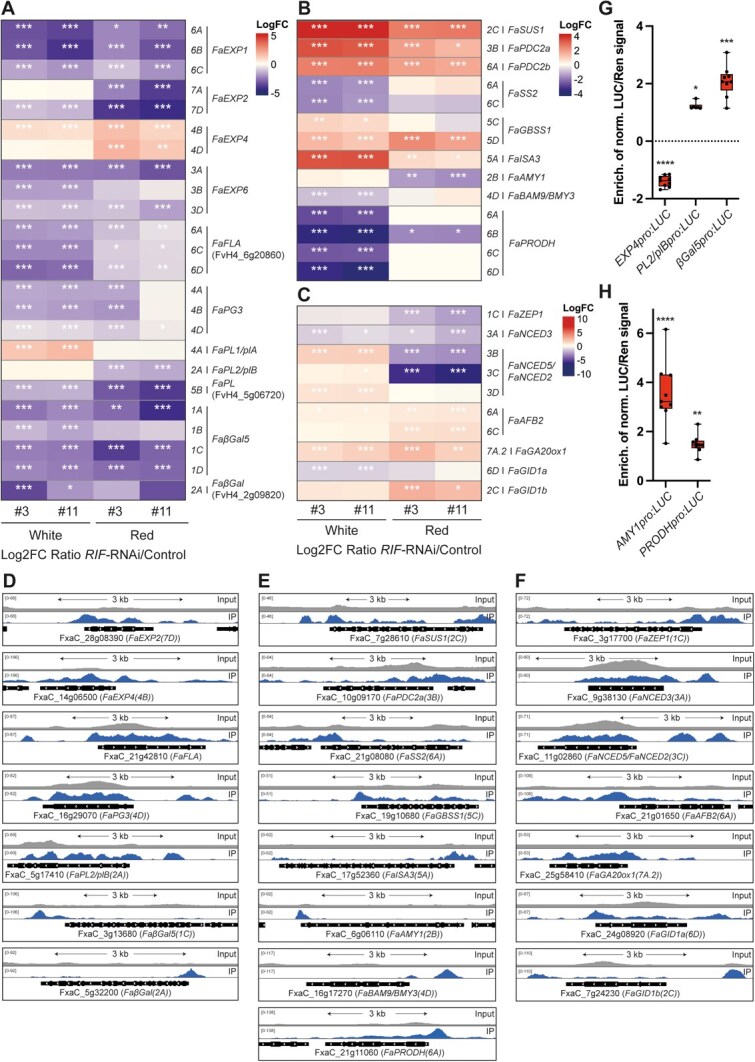
FaRIF directly regulates genes involved in cell wall modification, carbohydrate metabolism, and hormone biosynthesis and signaling pathways. (**A**) to (**C**) Heatmaps displaying the expression of FaRIF direct regulated genes related to cell wall modification (**A**), carbohydrate metabolism (**B**), and hormone biosynthesis and signaling pathways (**C**). Significant DEGs are marked with asterisks (^***^FDR ≤ 0.0005; ^**^FDR ≤ 0.005; ^*^FDR ≤0.05). (**D**) to (**F**) ChIP-seq peaks showing FaRIF binding sites and input reads over representative homoeologs of direct targets in cell wall modification (**D**), carbohydrate metabolism (**E**), and hormone-related genes (**F**). Detailed information is available in Table S6. (**G**, **H**) Direct regulation of *FaEXP4*, *FaPL2/plB*, and *FaβGal5* (**G**) and *FaAMY1* and *FaPRODH* (**H**) by FaRIF in a dual-luciferase reporter assay in infiltrated *N. benthamiana* leaves. Firefly LUCIFERASE (LUC) activity is presented relative to RENILLA (REN) activity and normalized against the control (empty effector vector). Statistically significant activation or repression of target genes is indicated by asterisks (^****^P ≤ 0.0001; ^***^P ≤ 0.005; ^**^P ≤ 0.01; ^*^P ≤ 0.05). Each value represents one biological replicate, each with 3 technical replicates.

Within the ‘Cell wall organization’ functional category enriched among the DEGs and FaRIF-direct targets, numerous genes encoding Expansins (EXP) proteins involved in cell wall loosening [71, 72], were mainly downregulated at one or both ripening stages*.* Notably, all homoeologs of *FaEXP1* and some of the most highly expressed *EXPs* during ripening in *F. vesca* [73] and *F.* × *ananassa*, as *FaEXP2(7D)*, the most expressed *EXP* gene, were identified as direct targets of FaRIF (Figs 5A and D, S7, and Table S6), supporting its direct role in promoting cell wall modification to favor strawberry fruit softening.

In addition to Expansins, nine Fasciclin-like arabinogalactan (FLAs)-encoding genes, involved in cell adhesion, were significantly downregulated, with homoeologs of *FaFLA17* (orthologous to FvH4_6g20860 in *F. vesca*) identified as direct FaRIF targets (Figs 5A and D, S7, and Table S6). Multiple genes encoding pectin-modifying enzymes were also primarily downregulated, including nine pectin methylesterases (*FaPMEs*) such as *FaPME39*, which promotes fruit softening in *F. vesca* [74] (Fig. S7 and Table S6). Furthermore, most homoeologs of the polygalacturonase-encoding gene *FaPG3* and homoeologs of the pectate lyases *FaPL1/plA* and *FaPL2/plB*, known regulators of strawberry fruit softening [42, 51, 75, 76], together with two additional *FaPL* genes, were identified as FaRIF direct targets (Figs 5A and D, S7, and Table S6).

FaRIF also directly targeted genes encoding rhamnogalacturonan-I-modifying enzymes, including homoeologs of four *β-galactosidase* (*βGal*) genes, such as *FaβGal1* and *FaβGal3* [77] (Table S6). Of particular interest, all homoeologs of a previously uncharacterized β-galactosidase-encoding gene, *FaβGal5* (orthologous to FvH4_1g12350 in *F. vesca*), contained FaRIF binding sites, suggesting conserved direct regulation. In addition, two other *FaβGal* genes, including *FaβGal4*, which promotes strawberry fruit softening [78], were indirectly regulated by FaRIF (Figs 5A and D, S7, and Table S6).

To further support the direct regulation of cell wall-related genes by FaRIF, we tested the promoters of *FaEXP4*, *FaPL2/plB*, and *FaβGal5* in transactivation assays. Consistent with their expression patterns in the RNAi lines, the *FaEXP4* promoter was negatively regulated, while those of *FaPL2/plB*, and *FaβGal5* were activated by FaRIF, validating them as direct functional targets of FaRIF (Fig. 5G).

Altogether, these data highlight FaRIF’s role in regulating cell wall-related genes critical for promoting fruit softening during strawberry fruit ripening, consistent with the increased fruit firmness observed in *RIF*-silenced and knockout lines in *F.* × *ananassa* and *F. vesca*, respectively [35, 36].

### FaRIF regulates primary metabolism

The ‘Carbohydrate metabolism’ and ‘Amino acid metabolism’ categories were significantly enriched among the DEGs and FaRIF-direct targets. Within carbohydrate metabolism, several genes involved in local sucrose turnover [79] were regulated by FaRIF (Figs 5B and E, S8A, and Table S6). Several *SUCROSE SYNTHASE1* (*FaSUS1*) homoeologs, which degrade sucrose into UDP-glucose and fructose, were upregulated at both ripening stages, with one containing a FaRIF binding site. In contrast, the only expressed *SUCROSE PHOSPHATE SYNTHASE1* (*FaSPS1*) homoeolog, *FaSPS1(2B)*, involved in sucrose biosynthesis, was downregulated at both stages. This pattern aligns with the altered sugar content in the *FaRIF*-RNAi receptacles, which accumulate more glucose and fructose but less sucrose than the control [36].

Fruit starch metabolism is also important for the final sugar content in ripe strawberries [80], with starch being progressively degraded by amylases during ripening. Thus, homoeologs of *STARCH SYNTHASE 2* (*FaSS2*), *GRANULE BOUND STARCH SYNTHASE 1* (*FaGBSS1*)—involved in amylose and starch biosynthesis in peas and Arabidopsis [81, 82]—and several genes encoding α- and β-amylases, as well as the isoamylase *FaISA3*, were differentially expressed, with some identified as FaRIF-direct targets (Figs 5B and E, S8A, and Table S6). Among them, *FaAMY1(2B)*, downregulated at the red stage and containing a FaRIF binding site, showed FaRIF-dependent transactivation (Fig. 5B, E, and  H; Table S6). These results support a role for FaRIF not only in regulating sugar content during strawberry ripening by regulating sucrose metabolism, as previously reported [35, 36], but also in controlling starch metabolism, contributing to the final sugar content in ripe strawberry fruits.

Additionally, MapMan analysis of the DEGs confirmed the enrichment of fermentation-related genes at both ripening stages, consistent with previous observations [36]. Two *PYRUVATE DECARBOXYLASE* genes (*FaPDC*s) and most homoeologs of two *ALCOHOL DEHYDROGENASE* genes (*FaADHs*) were upregulated at both stages in the *FaRIF-*silenced fruits, with one homoeolog of each *FaPDC* identified as a direct target. These findings support a direct role of FaRIF in modulating the aerobic/anaerobic balance, which is known to shift during strawberry fruit ripening [37].

Finally, we observed a notable enrichment among the FaRIF direct targets of genes involved in the degradation of proline (Pro), a common compatible osmolyte in plants [83] (Figs 3D, S4, and Table S5). Interestingly, all four homeologs of a *PROLINE DEHYDROGENASE* (*FaPRODH*) gene were identified in our ChIP-seq dataset and were significantly downregulated at the white stage in both *FaRIF*-RNAi lines (Figs 5B and E, S9, and Table S6), suggesting positive regulation by FaRIF, as further supported by the transactivation assay on the *FaPRODH(6A)* promoter (Fig. 5H). These results indicate a role for FaRIF in regulating Pro catabolism during ripening.

### FaRIF regulates phytohormone biosynthesis and signaling

Within the ‘Phytohormone action’ MapMan category, the ABA-related subcategory was enriched in *FaRIF*-RNAi fruits at the ripe stage and among the FaRIF direct targets (Fig. 3D). Thus, genes involved in the rate-limiting steps of the ABA biosynthetic pathway, such as *zeaxanthin epoxidase 1* (*FaZEP1*), and *9-cis-epoxycarotenoid dioxygenases* (*FaNCEDs*), were identified as FaRIF-direct targets and, along with *neoxanthin synthase* (*FaNSY*), were significantly downregulated in the *FaRIF-*silenced fruits (Figs 5C, F, S8B, and Table S6). Furthermore, homoeologs of ABA receptors such as *FaPYL2* (PYRL/PYL) and *FaABAR* (ABA receptor/Mg-chelatase H subunit (ABAR/CHLH)), both positive regulators of strawberry fruit ripening [11, 84–86], were positively regulated by FaRIF, with one of the *FaABAR* homoeologs identified as a direct target (Fig. S8B, and Table S6). Additionally, protein kinase-encoding genes *SUCROSE NONFERMENTING1-RELATED PROTEIN KINASE 1*, (*FaSnRK1s*), involved in ABA signaling [87], were found enriched among the FaRIF targets and differentially expressed (Figs 3D, S4B, S8B, and Table S6). All these findings are consistent with the altered ABA content and delayed ripening phenotype of *FaRIF-*silenced fruits [36], supporting the positive role of FaRIF in regulating the key phytohormone involved in strawberry fruit ripening [11].

Auxins and gibberellins are crucial for promoting strawberry fruit growth and inhibiting ripening [6] by suppressing ABA accumulation through *CYP707A4a* expression activation [16]. Interestingly, genes related to the content and signaling pathways of these phytohormones were misregulated in the *FaRIF*-silenced fruits, with several identified as FaRIF-direct targets (Figs 5C and F, S8B, and Table S6). Among them, most homoeologs of the auxin receptor gene *AUXIN SIGNALING F-BOX 2* (*FaAFB2*), which positively mediates auxin signaling in Arabidopsis [88], were upregulated at the red stage, while the negative regulator *FaAUX/IAA13* was indirectly repressed at the white stage, suggesting a negative role of FaRIF in regulating auxin signaling. FaRIF may also repress the GA biosynthetic gene *FaGA20ox1*, and indirectly misregulate the GA catabolic gene *FaGA2ox1* [89, 90]*.* Additionally, homoeologs of the GA signaling genes *GIBBERELLIN-INSENSITIVE DWARF1* (*FaGID1a* and *FaGID1b*) [89] were identified as FaRIF-direct targets. These results suggest that, in addition to regulating ABA, FaRIF may influence strawberry fruit ripening by modulating auxin and GA biosynthesis and signaling pathways.

### FaRIF regulates genes involved in secondary metabolism

The ‘secondary metabolism’ bin was also enriched at the red stage in the *FaRIF-*silenced lines (Fig. 3D). Notably, several genes involved in terpenoid biosynthesis were identified, including homoeologs involved in the first committed steps in the mevalonate (MVA) and the methylerythritol phosphate (MEP) pathways, specifically, 3-hydroxy-3-methylglutaryl-CoA reductases (HMGRs) and 1-deoxy-D-xylulose 5-phosphate synthase (DXS), respectively. These genes were indirectly misregulated in a manner where those normally induced during ripening were downregulated, while those typically repressed during this process were upregulated (Fig. S10 and Table S6). Additionally, two homoeologs of a terpene synthase, *FaTPS11* [91], were identified as FaRIF-direct targets and downregulated at the ripe stage in the *FaRIF-*silenced fruits. Moreover, the four homoeologs of the *PHYTOENE SYNTHASE 1* (*FaPSY*), which initiates the carotenoid biosynthesis, were also downregulated at the red stage, with two being FaRIF direct targets. These findings suggest a novel role of FaRIF in the regulation of terpenoid biosynthesis during strawberry ripening.

**Figure 6:**
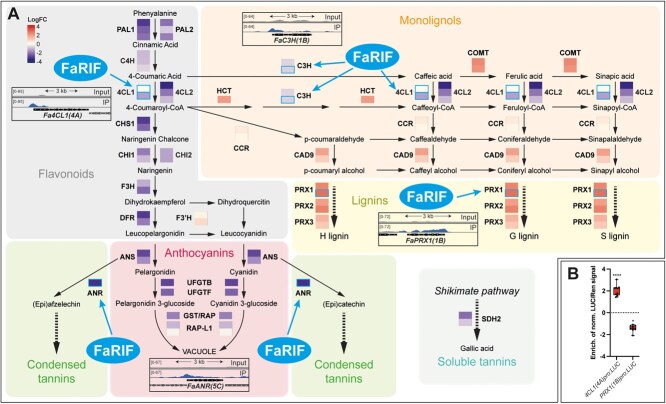
FaRIF indirectly and directly regulates genes involved in the phenylpropanoid, flavonoid, monolignol, lignin, condensed and soluble tannins pathways. (**A**) Biosynthetic pathways with enzymatic steps colored according to the average log2 expression ratio of *35Spro:RIF*-RNAi vs. control for differentially expressed homoeologs (genes in bold) in both transgenic lines at the red stage. Red and purple indicate upregulation and downregulation, respectively, in both *FaRIF-*silenced lines. Direct targets of FaRIF are depicted with a blue frame, with ChIP-seq peak insets showing FaRIF binding sites. Detailed information is available in Table S6. (**B**) Direct regulation of *Fa4CL1(4A)* and *FaPRX1(1B)* by FaRIF in a dual-luciferase reporter assay in infiltrated *N. benthamiana* leaves. Firefly LUCIFERASE (LUC) activity is presented relative to RENILLA (REN) activity and normalized against the control (empty effector vector). Each value represents one biological replicate, each with 3 technical replicates. Statistically significant activation or repression of target genes is indicated by asterisks (^****^P ≤0.0001; ^*^P ≤ 0.05).

A specific MapMan bin related to phenolic compounds was also enriched at the red stage, although only a few were identified as direct FaRIF targets (Figs 6, S11, and Table S6). Most structural genes in the phenylpropanoid, flavonoid, and monolignol pathways, as well as *Reduced Anthocyanins in Petioles-Like 1* (*FaRAP-L1*), an anthocyanin transporter promoting strawberry fruit pigmentation [92], appeared to be indirectly regulated. Among the direct targets, we identified all homoeologs of *4-Coumaroyl-CoA ligase 1* (*Fa4CL1*), along with some homoeologs of *4-Coumarate 3-hydroxylase* (*FaC3H*) and a peroxidase-encoding gene (*FaPRX1*). The direct regulation of *Fa4CL1(4A)*, and *FaPRX1(1B)* were validated by LUC assay (Fig. 6B). Together, these results are consistent with previous findings showing that FaRIF positively regulates anthocyanin content (Fig. 1C) [35, 36], while repressing lignification [36], thereby contributing to fruit coloration and softening during strawberry ripening.

### FaRIF forms homo- and heterodimers with NAC TFs and other proteins

Our transcriptomic and ChIP-seq data highlighted the central role of FaRIF in multiple processes essential for fruit ripening. Because NAC proteins are known to modulate their activity through homo- or heterodimerization and interactions with other protein partners, we investigated potential FaRIF-interacting proteins using a TurboID-based proximity labeling approach. We first validated the nuclear localization of the *FaRIF-TurboID-GFP* fusion protein by transient expression in *N. benthamiana* leaves (Fig. S12A). Then, to identify proteome-wide interactors of FaRIF, three biological replicates of samples expressing *FaRIF-TbID* were compared with those expressing the negative control construct (Fig. S12C). After removing proteins also present in the control, we confidently identified a total of 973 peptides corresponding to 43 protein groups, representing 247 unique proteins that were exclusively present in the *FaRIF-TbID* samples across the three biological replicates (Table S7). Interestingly, several NAC TFs close to FaRIF, which identified peptides are common for all of them, were found among the potential interactors, including FaRIF itself as well as FaNAC004, FaNAC015, FaNAC021, FaNAC034, FaNAC039, FaNAC040, FaNAC045, FaNAC064, and FaNAC077 (Table S7), suggesting that FaRIF may form both homo- and heterodimers with other close NAC family members.

In addition, other putative interactors included several ribosomal and RNA-binding proteins. Among these was the putative orthologue of Arabidopsis PUF RNA-binding protein PUMILIO2 (PUM2), involved in translational regulation, mRNA localization [93], and preribosomal RNA processing [94, 95], as well as CTC-Interacting Domain 4 (CID4), which is involved in developmental pathways and stabilizes target mRNAs via 3′UTR binding [96]. Several YTH domain-containing proteins, including FaYTH3, FaYTH4, FaYTH6, and FaYTH8, orthologs of *F. vesca* proteins recently identified [97] and reported to regulate mRNA splicing, processing, stability, and translation [98], were also identified.

Proteins related to protein folding and processing also emerged as FaRIF interactors, notably CCT3, a component of the chaperonin TCP-1 ring complex (TRiC/CCT) essential for the proper folding of specific eukaryotic proteins [99], and UBIQUITIN-SPECIFIC PROTEASE 7 (UBP7), which cleaves ubiquitin chains to likely prevent substrate degradation [100, 101], suggesting a role for these proteins in FaRIF protein homeostasis. Finally, we identified two Ca^2+^-dependent lipid-binding (CalB) proteins, which generally bind calcium and phospholipids [102], specifically FaCalB1 and FaCalB2, each containing a single C2 domain.

**Figure 7:**
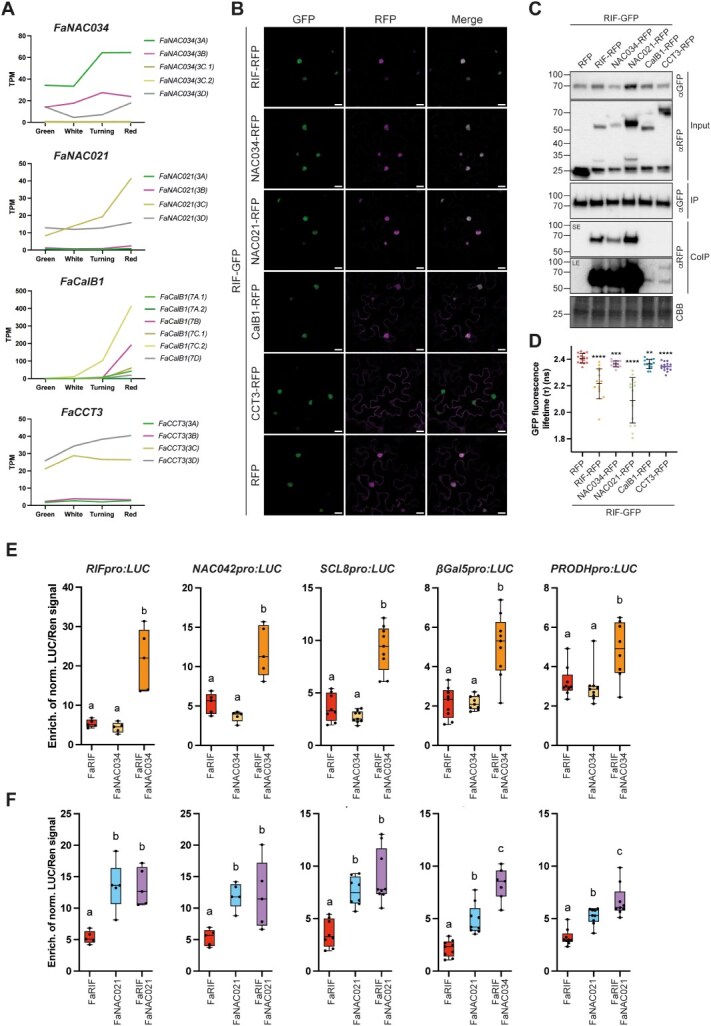
Validation of FaRIF interactions with candidate protein partners. (**A**) Expression patterns of the homoeologs of candidate FaRIF interactors in *F.* × *ananassa* receptacles at four ripening stages. Data adapted from Sánchez-Sevilla et al. [49] and Liu et al. [46]. (**B**) Subcellular localization of FaRIF-GFP and RFP-tagged candidate interactors in *N. benthamiana* leaves. Confocal images were captured 48 h post-infiltration. Scale bars = 20 μm. (**C**, **D**) Co-immunoprecipitation (Co-IP) and FRET-FLIM assays of FaRIF-GFP with RFP-tagged proteins: FaRIF, FaNAC034, FaNAC021, FaCalB1, FaCCT3, and free RFP (negative control). All tagged proteins were transiently coexpressed in *N. benthamiana* leaves. (**C**) FaRIF-GFP was immunoprecipitated using anti-GFP-Trap beads. Total protein (input), IP, and Co-IP samples were analyzed by immunoblotting. Equal loading was confirmed by Coomassie Brilliant Blue (CBB) staining of input samples. GFP- and RFP-tagged proteins were detected using anti-GFP (αGFP) and anti-RFP (αRFP) antibodies, respectively. SE and LE refer to short and long exposure times, respectively. Predicted protein sizes: RFP: 25.43 kDa; RIF-GFP: 66.69 kDA; RIF-RFP: 65.12 kDa; NAC034-RFP: 66.47 kDa; NAC021-RFP: 66.53 kDa; CalB1-RFP: 54.89 kDa; CCT3-RFP: 87.36 kDa. (**D**) FRET-FLIM in 15 nuclei from five independent plants per assay. Statistically significant protein interaction measured by FRET-FLIM is indicated by asterisks (^****^P ≤ 0.0001; ^***^P ≤0.005; ^**^P ≤ 0.01). (**E**, **F**) Dual-luciferase reporter assays testing activation of the *FaRIF*, *FaNAC042*, *FaSCL8*, *FaβGal5*, and *FaPRODH* promoters in *N. benthamiana* leaves. The individual activity of FaRIF and its combined activity with FaNAC034 (**E**) and FaNAC021 (**F**) were tested. Firefly LUCIFERASE (LUC) activity is presented relative to RENILLA (REN) activity and normalized against the control (empty effector vector). Each value represents the mean of 3 technical replicates from 5–9 biological replicates ±SD. Different letters indicate significant differences at *P* ≤ 0.05.

We next selected several of these interactors identified by proximity labeling for further validation, focusing on FaRIF itself, as well as FaNAC021 and FaNAC034. Both NAC proteins are encoded by homoeologs upregulated throughout ripening, suggesting their potential involvement in this process (Fig. 7A). Additionally, we included C2 domain protein FaCalB1, whose expressed homoeologs all correlate with *FaRIF* expression, and the chaperonin FaCCT3 (Fig. 7A). Transient expression of RFP-tagged versions of these putative interactors in *N. benthamiana* leaves confirmed the colocalization with FaRIF-GFP in nuclei, except for FaCalB1, which also exhibited cytoplasmic localization, and FaCCT3, which was predominantly localized in the cytoplasm with limited nuclear presence (Fig. 7B). Co-immunoprecipitation (Co-IP) and Förster resonance energy transfer-fluorescence lifetime imaging (FRET-FLIM) assays subsequently confirmed the interactions between FaRIF and all selected candidates (Fig. 7C and D). Notably, FaRIF homo- and heterodimerization with FaNAC021 and FaNAC034 showed the strongest interactions, while weaker interactions were detected with FaCalB1 and FaCCT3, despite FaCCT3’s low nuclear signal (Fig. 7C and D). These results confirm that these proteins identified *in vivo* via proximity labeling interact with FaRIF and may contribute to the regulation of its activity and/or protein homeostasis.

### FaRIF activity is modulated by its interactors FaNAC021 and FaNAC034

Our previous data confirmed that FaRIF can form both homo- and heterodimers, at least with FaNAC021 and FaNAC034. Thus, we next investigated whether the formation of these heterodimers influences FaRIF’s transcriptional activity on specific target genes. To this end, we selected five validated FaRIF-bound promoters (its own promoter, *FaNAC042*, *FaSCL8*, *FaβGal5*, and *FaPRODH*) and assessed the transcriptional activation mediated by FaNAC021 and FaNAC034 using dual-luciferase transactivation assay in *N. benthamiana* leaves (Fig. 7E and F)*.* Interestingly, FaNAC034 activated all five promoters to levels comparable to FaRIF (Fig. 7E), whereas FaNAC021 induced significantly higher expression than FaRIF for each promoter tested (Fig. 7F). To explore the potential combinatorial effects of heterodimerization on the activation of these promoters, we coinfiltrated FaRIF with either FaNAC034 or FaNAC021. Notably, coexpression of FaRIF and FaNAC034 led to greater activation than either TF alone across all tested promoters, suggesting a synergistic effect of this heterodimer. This effect was also observed for the *FaβGal5* and *FaPRODH* promoters when FaRIF was coexpressed with FaNAC021. In contrast, no further activation was detected for the *FaRIF*, *FaNAC042*, and *FaSCL8* promoters upon coexpression of FaRIF and FaNAC021, indicating that FaRIF does not enhance the transcriptional activity of FaNAC021 on these promoters. These results suggest that the outcome of FaRIF-FaNAC021, and probably FaRIF-FaNAC034 heterodimerization may depend on the specific promoter context. Further experiments are needed to understand the broader functional relevance of FaRIF heterodimerization and the extent of target genes overlap among these three NAC TFs genome-wide.

## Discussion

In this study, we expand the functional characterization of the NAC TF RIF in the cultivated octoploid strawberry, uncovering new regulatory layers not addressed in previous works. By developing optimized protocols for both ChIP-seq and TurboID proximity labeling, we provide a comprehensive and fruit-specific view of the FaRIF regulatory network.

Building upon prior analyses performed in *F. vesca*, we first asked how the complexity of the octoploid genome influences FaRIF regulation and function. *RIF* expression increases throughout ripening in both *F.* × *ananassa* and *F. vesca* receptacles [35, 36]. However, RNA-seq data from receptacles at four ripening stages mapped to the *F.* × *ananassa* reference genome [46] revealed that *FaRIF(3D)*, encoded by the *F. viridis* subgenome, did not follow this trend, while *FaRIF(3A)*, from the *F. vesc*a subgenome, was the most expressed homoeolog (Fig. 1B). ChIP-seq data revealed FaRIF’s preferential binding to the *F. vesca* subgenome (Figs 2E and S3), consistent with its dominance in *F.* × *ananassa* due to lower transposable elements density and higher homoeologs expression [30]. Nevertheless, exceptions exist. For instance, the most expressed homoeolog of *FaEXP1*, *FaPME39, FaNCED5/FaNCED2*, and *FaCHS1* is encoded by another subgenome, following also their respective homoeologs different expression trends (Table S6). Thus, although the extended use of the diploid *F. vesca* genome as the reference for transcriptome analysis in the octoploid species has proven successful and informative, the availability of a high-quality reference genome for *F.* × *ananassa* marks a significant advancement. Despite the analytical challenges posed by the complexity of the octoploid genome, including the presence of homoeologs for each gene, the use of the *F.* × *ananassa* genome provides a more accurate and comprehensive view of the transcriptomic landscape in this economically valuable crop. It is also important to note that although overexpression of TFs can modify protein stoichiometry and potentially alter the number of detected targets, this strategy remains the most feasible in octoploid strawberry, where the generation of complemented knockout lines is technically challenging and the production of high-quality ChIP-grade antibodies against native TFs is often unfeasible. Despite these potential caveats, our ChIP-seq dataset is supported by integration with RNA-seq data and experimental validation of selected targets, providing robust evidence for FaRIF’s *in vivo* binding profile.

With this genomic framework in mind, we next investigated the extent to which *FaRIF* directly regulates ripening-related processes. Combined DEG and ChIP-seq analyses revealed that FaRIF directly or indirectly regulates multiple pathways central to ripening, including ABA biosynthesis and signaling, sugar and proline metabolism, secondary metabolites biosynthesis, cell wall remodeling, and energy balance. Among the DEGs in *FaRIF-*RNAi lines, 15.3% were identified as direct targets, consistent with findings for its *F. vesca* ortholog (Figs 3C and S5A) [35]. These results underline FaRIF’s central role as an upstream regulator, acting directly or through the regulation of other TFs such as FaSPT and FaSCL8. Notably, FaRIF directly regulates genes involved in ABA biosynthesis and signaling, the primary hormone modulating strawberry fruit ripening [10–12]. The enrichment of ABA signaling-related SnRK1-encoding genes among its direct targets further supports this central function, consistent with their roles in ABA-mediated seed maturation in pea [103], as well as sugar accumulation in strawberry [79]. This underscores the critical role of FaRIF orchestrating ripening, as evidenced by the complete ripening block in *Fvrif* knockout *F. vesca* mutants [35].

Our results show that FaRIF regulates sugar metabolism not only by modulating sucrose-related genes, but also by potentially controlling starch metabolism. Moreover, we found that FaRIF may play a role in the reported decline in proline content during ripening [104], as we found that FaRIF directly activates the expression of the catabolic gene *FaPRODH*. Because the expression of the homeologs of this gene peak at the white stage, with *FaPRODH(6A)* and *FaPRODH(6B)* being the most highly expressed (Fig. S9B) [46, 49], their downregulation in the *FaRIF-*RNAi lines may explain their increased Pro accumulation observed at the red stage [36] (Fig. S9C). It has been proposed that the increase in total soluble solids during strawberry fruit development and ripening leads to a decrease in water potential, which may act as a primary signal initiating the ripening process [105]. However, the elevated Pro levels observed in *FaRIF*-silenced fruits at the red stage would be expected to further lower the osmotic potential, which is not in line with their delayed ripening phenotype. Therefore, further studies are required to better understand the proposed role of osmotic potential in ripening initiation and to clarify the specific contribution of Pro to this process. Nevertheless, given its involvement in redox homeostasis, mitochondrial energy metabolism, and broader metabolic signaling [106], Pro may influence ripening through additional mechanisms beyond its contribution to osmotic potential.

In addition to primary metabolism, FaRIF influences specialized metabolic pathways. For instance, it directly regulates key genes involved in flavonoid and lignin biosynthesis, such as *Fa4CL1*, *FaANR*, *FaC3H*, and *FaPRX1*. Interestingly, while DAP-seq analysis identified *FvMYB10* as a direct target of FvRIF in *F. vesca* [35], this regulation was not observed in *F.* × *ananassa*, which may reflect species-specific regulatory differences, with a stronger impact of RIF on anthocyanin biosynthesis in *F. vesca* than in *F.* × *ananassa*, or a false-negative in our ChIP-seq. Furthermore, FaRIF appears to regulate terpenoid biosynthesis, including VOCs and carotenoids. Among the direct targets, we identified *FaTPS11*, the closest paralog to *NEROLIDOL SYNTHASE1* (*FaNES1*), which is involved in the biosynthesis of the monoterpene linalool and the sesquiterpene nerolidol [107], VOCs that contribute sweet and flowery notes to strawberry aroma [108]. While *FaTPS11* has not yet been functionally characterized*,* its homoeologs were identified as candidate genes for the biosynthesis of these VOCs in a multiomic QTL analysis [109]. We therefore hypothesize that FaRIF directly contributes to aroma formation by promoting *FaTPS11* expression.

Remarkably, our ChIP-seq study has allowed the identification of new FaRIF targets not previously reported in the DAP-seq analysis in *F. vesca* (Table S1; Fig. S5B), including FaRIF itself, which suggests a feedback positive regulatory loop. We also identified other novel FaRIF targets in our dataset, including the ripening-related TFs *FaSCL8* and *FaGLK2*, genes involved in ABA biosynthesis and signaling, (*FaZEP1*, *FaNCED3*, *FaNCED5*, *FaHY5*, *FaBBX19*, *FaHVA22*, *FaSnRK2.6*), cell wall modification (*FaEXP1*, *FaEXP4*, *FaβGal5*, *FaPG3*, *FaPL1/plA*, *FaPL3*), and in quality traits related to sugar, ascorbic acid, and VOC content (*FaSUS1*, *FaAMY1*, *FaVTC2*, *FaDOF2*)*.* These findings highlight the value of *in vivo* binding site identification through ChIP-seq.

Having defined FaRIF’s transcriptional network, we next investigated how FaRIF’s activity might be modulated through protein–protein interactions. NAC proteins are known to modulate their activity by forming homo- or heterodimers [50, 110–114], and by interacting with other proteins, such as bZIP-type [115] and homeodomain (HD) TFs [116], as well as enzymes like phosphatases [117] and kinases [35]. This prompted us to explore FaRIF’s protein interaction network through TurboID in strawberry fruits. Interestingly, ripe strawberry fruits naturally contain high biotin levels (~15 ng/g fresh weight; [118]), unlike the commonly used model species for proximity labeling assays, *N. benthamiana* and *A. thaliana*. Consistent with this, no significant differences in global protein biotinylation were observed between fruits with or without exogenous biotin supplementation (Fig. S12B).

This assay showed that FaRIF interacted with several NAC TFs, including FaRIF itself, FaNAC021, and FaNAC034. Co-IP and FRET-FLIM validations confirmed the formation of both homo- and heterodimers. Notably, FaRIF heterodimerization with both FaNAC034 and FaNAC021 resulted in a synergistic activation of all tested promoters in the case of FaNAC034, and of two out of five promoters in the case of FaNAC021. Similar synergistic effects among NAC TFs have been previously reported, such as in grapevine, where combinations of VviNAC03, VviNAC33, and VviNAC60 enhance transcription of genes involved in chlorophyll degradation and anthocyanin accumulation [110]. Likewise, in *N. tabacum*, NtNAC028 and NtNAC080 synergistically promote jasmonic acid biosynthesis by coregulating *NtLOX3* expression [112]. Further studies will be needed to determine the extent of coregulated targets shared by FaRIF, FaNAC021, and FaNAC034, and to understand how FaRIF’s interactions with these NAC TFs contribute to fine-tune target gene expression during strawberry fruit ripening.

In addition to NAC TFs, our TurboID-based proximity labeling analysis revealed ribosomal proteins as potential interactors. While these may reflect biotinylation during translation of the FaRIF-TbID protein, functional complexes with these proteins cannot be ruled out. Interestingly, FaRIF was found to interact with RNA-binding proteins, including PUM2, which is involved in translational regulation, mRNA localization, and preribosomal RNA processing in Arabidopsis [93–95]. PUM proteins have not been studied in fruit ripening, but related proteins such as APUM5 and APUM9 modulate ABA-responsive genes [119, 120]. This raises the possibility that FaRIF-PUM interactions contribute to ABA signaling regulation in strawberry ripening, a hypothesis for further exploration.

Other identified RNA-binding proteins included YTH domain-containing proteins, which recognize N6-methyladenosine (m^6^A) modifications in mRNAs and influence their stability and translation [19, 98]. In strawberry, changes in m^6^A methylation occur at the onset of strawberry ripening, stabilizing ABA-related mRNAs that encode the rate-limiting enzyme *NCED5* and the TF *AREB1*, while also facilitating the translation of *ABAR*, an ABA receptor [19]. SlYTH1 and SlYTH2*,* orthologs in tomato of the FaRIF interactors FaYTH8 and FaYTH3, respectively, have been implicated in the development and ripening of the fruit [121, 122]. Notably, *SlYTH2* knockout mutants display delayed internal fruit ripening and altered ABA content [121]. Furthermore, two homoeologs of a closely related gene, *FaYTH4*, coexpressed with FaRIF (Table S7). This suggests that FaRIF, in addition to regulating the expression of ABA-related genes, may stabilize their mRNAs in conjunction with FaYTH proteins, integrating transcriptional and post-transcriptional regulation during ripening.

Proteins involved in protein folding and processing were also identified as FaRIF interactors. Among them, the chaperonin complex TriC/CCT subunit CCT3 was validated as an interactor (Fig. 7C). The TriC/CCT complex assists in folding specific eukaryotic proteins [99] and has been reported to directly regulate HSF1, a stress-responsive TF [123]. Whether the interaction between FaRIF and CCT3 serves a ripening-specific function remains to be elucidated.

Finally, two Ca^2+^-dependent lipid-binding proteins containing a single C2 domain, FaCalB1 and FaCalB2, were identified, with FaCalB1 validated by Co-IP (Fig. 7C). Calcium acts as an intracellular messenger in ABA signaling [124] and stimulates flavonoid-related genes and anthocyanin accumulation in strawberry [125]. Single C2 domain proteins such as the Arabidopsis CalB1 orthologue, and a closely related protein in rice, *OsERG1*, are involved in plant defense signaling [126, 127]. Interestingly, differences in the membrane binding capability and subcellular localization have been found in close homologs of FaCalB1 in rice [127, 128] and barley [129], being the latter localized in nuclei as FaCalB1 (Fig. 7B). It is intriguing to speculate that FaCalB proteins participate in calcium-mediated ABA signaling and ripening progression in coordination with FaRIF, a hypothesis that requires further investigation.

Altogether, our integrative analysis positions FaRIF as a key regulator of strawberry fruit ripening, acting through a combination of direct transcriptional control and diverse protein–protein interactions. While many genomic studies have described ripening-associated expression changes, our work advances understanding by providing an *in vivo* mapping of FaRIF binding sites in the octoploid genome and by establishing FaRIF’s interactome through proximity labeling. These findings reveal regulatory mechanisms—including subgenome-specific targeting, NAC dimer specificity, and potential coordination with mRNA stability and protein homeostasis pathways—that were not detectable through transcriptomics alone. Future studies should define the coregulatory mechanisms and functional implications of these interactions, and their contributions to ripening progression and fruit quality traits in strawberry.

## Material and methods

### Plant material and growth conditions

Stable *35Spro:FaRIF-RNAi*, *35Spro:FaRIF*, and *35Spro:FaRIF-GFP* transgenic lines were previously generated [36]. The full-length *FaRIF* constructs correspond to the *FaRIF(3B)* ortholog. *F.* × *ananassa* cv. Camarosa control and transgenic strawberry adult plants were grown and maintained in a shading house (IFAPA, Churriana, Málaga, Spain) and greenhouse (IHSM, Málaga, Spain) conditions.

### Transcriptome analysis by RNA-seq

RNA-seq data from *FaRIF*-RNAi receptacles [36] were remapped and annotated to the *F.* × *ananassa* cv. Camarosa genome assembly v1.0.a1 [30] and reference genome v1.0.a2 [46], respectively. This analysis was conducted using the European Galaxy server (https://usegalaxy.eu/) [130]. Sequence alignment was performed using the HISAT2 tool for sequence alignment, followed by read counting with the htseq-count tool. The edgeR tool was employed for differential expression analysis of count data. Genes with Log2(FC) ≤ −1 or ≥ 1 and FDR ≤ 0.05 were considered DEGs. Functional categorization was done using MapMan bins [52]. RNA-seq data are available at GEO under the accession number GSE167107.

### Protein structure prediction

Protein models of FvRIF and FaRIF were predicted using AlphaFold 3 [48]. The five models predicted were visualized and edited with PyMOL [131].

### Chromatin immunoprecipitation and ChIP-seq analysis

ChIP was performed using leaves from *35Spro:RIF-GFP* lines following a modified Gendrel et al. [132] protocol, with anti-GFP antibody (Abcam ab290) and subsequent qPCR validation on selected targets. Two biological replicates were sequenced on the Illumina NovaSeq6000 platform, and raw reads were processed with Trim Galore!, mapped to the *F. × ananassa* Camarosa genome using Bowtie2, and peaks were called with MACS2 (fold-change >2, FDR-adjusted *q* < 0.001). Overlapping peaks between replicates were identified with BEDTools. Further filtering used summit proximity (≤200 or ≤ 100 bp) and IDR ≤0.05 to define increasingly stringent VLs (VL1–VL3). Functional annotation of peaks and motif discovery were performed with ChIPseeker and MEME-ChIP, respectively. Peak distribution across subgenomes was evaluated by Chi-square tests. Full protocol and analysis details are provided in Supplementary Information.

ChIP-seq data are available at GEO under accession number GSE304403.

### TurboID-based proximity labeling and MS analysis plasmid construction

TurboID fusion constructs (*FaRIF-TbID-GFP* and *FaRIF-3xHA-TbID*) were generated using the Ti-TAN toolbox [133]. The latter was transiently expressed in white-stage fruits. Biotin-labeled proteins were enriched using streptavidin beads, and recombinant protein expression confirmed by immunoblotting. Enriched proteins were analyzed by LC–MS/MS on an Orbitrap QExactive HF system. Protein identification and quantification were performed using MaxQuant and Perseus software. The primers for the plasmids construction are listed in Table S8. Full protocol and parameters are provided in Supplementary Information.

### Agroinfiltration in *N. benthamiana*

For subcellular localization, protein–protein interaction, and reporter assays, constructs were introduced into *N. benthamiana* leaves by Agrobacterium tumefaciens (GV3101)-mediated infiltration. All assays used 3- to 4-week-old plants, a standard infiltration buffer (10 mM MES pH 5.6, 10 mM MgCl₂, 1 mM acetosyringone). Either a single TF construct was infiltrated at OD_600_ = 0.8, or two TF constructs were co-infiltrated at OD_600_ = 0.4 each, together with p19 at OD_600_ = 0.2 to suppress gene silencing. Plants were grown under long-day conditions (16 h light/8 h dark, 23°C), and samples were collected 2–3 days post-infiltration.

### Subcellular localization

To assess nuclear localization and colocalization, transient expression assays in *N. benthamiana* were performed using CDS of *FaRIF* and four candidate interactors (*FaNAC021*, *FaNAC034*, *FaCalB1*, *FaCCT-3*), cloned from cDNA prepared from red fruits of the octoploid *F.* × *ananassa* cv. Camarosa into the pGWB554 vector (mRFP tag). *FaRIF* was also cloned into pK7WG2D vector (GFP tag). The empty vector pGWB554 (*35Spro:mRFP*) was used as a control. Fluorescent signals were observed by confocal microscopy (ZEISS LSM 880). Excitation/emission settings were 488/500–550 nm for GFP and 543/600–650 nm forRFP. The primers used for plasmid construction are listed in Table S8.

### Co-immunoprecipitation assay

Total proteins were extracted from co-infiltrated leaves, and FaRIF-GFP complexes were immunoprecipitated using GFP-Trap magnetic beads (Chromotek). Interacting proteins were detected by immunoblotting using anti-GFP and anti-RFP antibodies. Full buffer compositions and washing steps are provided in Supplementary Information.

### FRET-FLIM imaging

For FRET-FLIM experiments, donor eGFP- and mRFP-tagged proteins cloned in pGWB554 were coexpressed in *N. benthamiana* leaves as previously described. Discs from transformed leaves were analyzed two days after agroinfiltration using a Leica STELLARIS 8 FALCON FLIM Microscope® confocal microscope system. eGFP and mRFP fluorophores were excited using a WLL laser at 488 nm and 561 nm, respectively, with emission spectra collected at 500–540 nm for eGFP and 600–630 nm for mRFP. Time-resolved fluorescence decay curves were acquired using time-correlated single-photon counting (TCSPC). FLIM data were analyzed with LAS X FLIM FCS software. The fluorescence lifetime (τ) values shown in the figures correspond to the average fluorescence lifetime of the donor and were collected from regions of interest (ROIs) encompassing the entire nuclear area in cells expressing both fluorophores. Mean lifetimes are presented as means ± SD.

### Dual-luciferase reporter assay

Dual-luciferase assays were performed in *N. benthamiana* leaves using the agroinfiltration procedure described above. Promoter sequences (ranging from 900 bp to ~2 kb upstream of the TSS) were cloned into the pGreenII 0800-LUC plasmid [134]. The genes whose promoters were cloned and their corresponding sizes are listed in Table S8. One or two TF constructs were co-infiltrated at a final OD_600_ of 0.4, along with their corresponding putative target promoter at a final OD_600_ of 0.4. In all mixtures, an *Agrobacterium tumefaciens* clone overexpressing the RNA silencing suppressor p19 was included at OD_600_ = 0.2. The pK7WG2 empty vector was used as a negative control. Each experiment included one infiltrated leaf per plant across 5 to 9 independent plants, with each leaf considered an independent biological replicate. Two 1 cm^2^ leaf fragments were collected per inoculation. Firefly luciferase and Renilla luciferase activities were measured 3 days post-infiltration using the Dual-Luciferase® Reporter Assay System (Promega) and a GloMax® Navigator luminometer (Promega). Data are expressed as the luciferase/renilla (LUC/REN) ratio. Primers used for plasmid construction are listed in Table S8.

## Supplementary Material

Web_Material_uhaf362

## Data Availability

The data that support the findings of this study are available in Figs S1–S12, Supplementary Dataset S1, and Tables S1–S8. RNA-seq data and ChIP-seq data are available at GEO under the accession numbers GSE167107 and GSE304403, respectively.
