# Supplementary figures and images for: FaRIF as a key regulator of strawberry fruit ripening: deciphering its targets and interaction networks
Source: Hortic Res. 2026 Jan 6;13(4):uhaf362. doi: 10.1093/hr/uhaf362 (PMC13150854; doi:10.1093/hr/uhaf362)

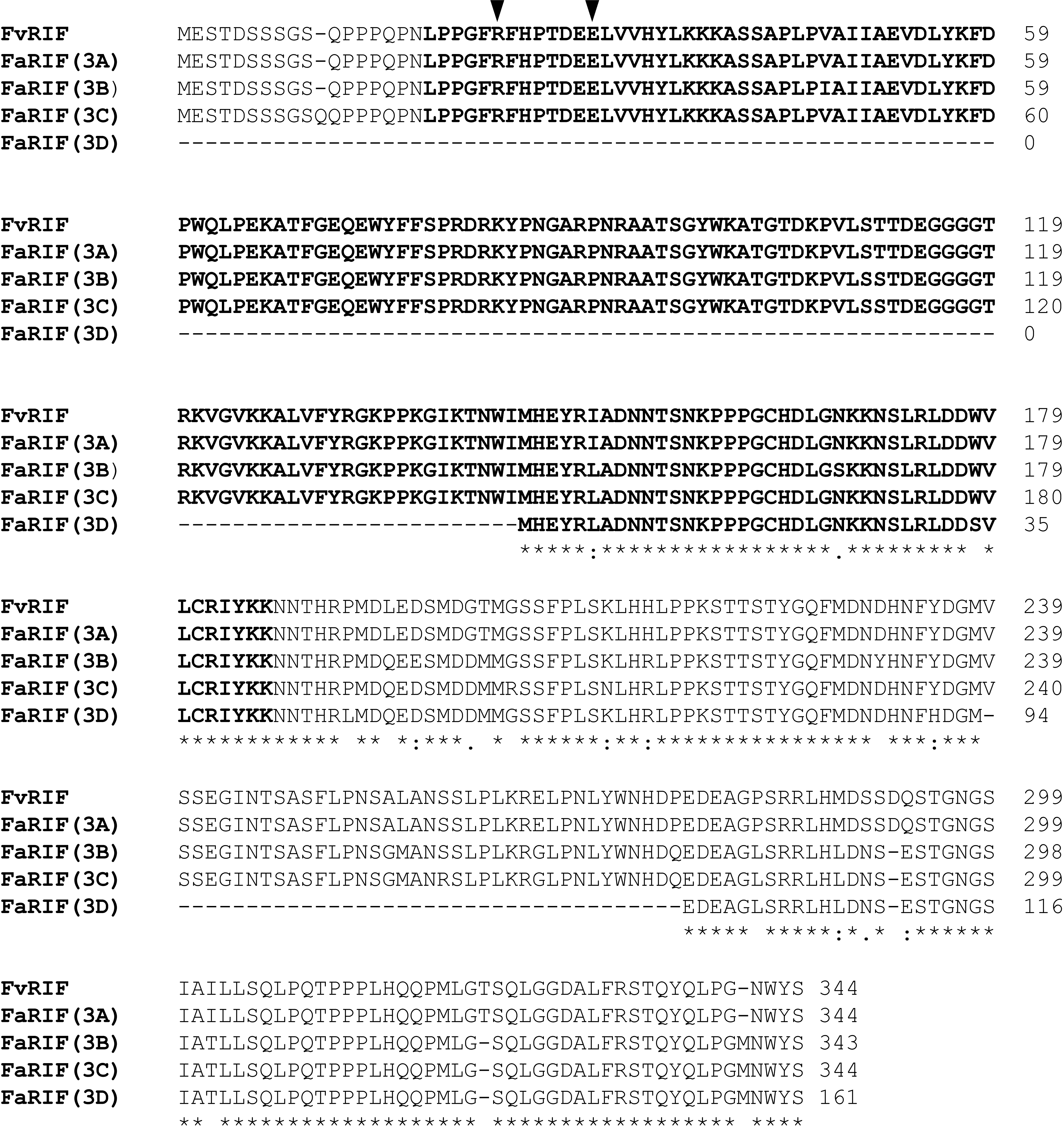

Supplement: Web_Material_uhaf362 [file web_material_uhaf362.zip › Supplementary Fig. S1.tif]

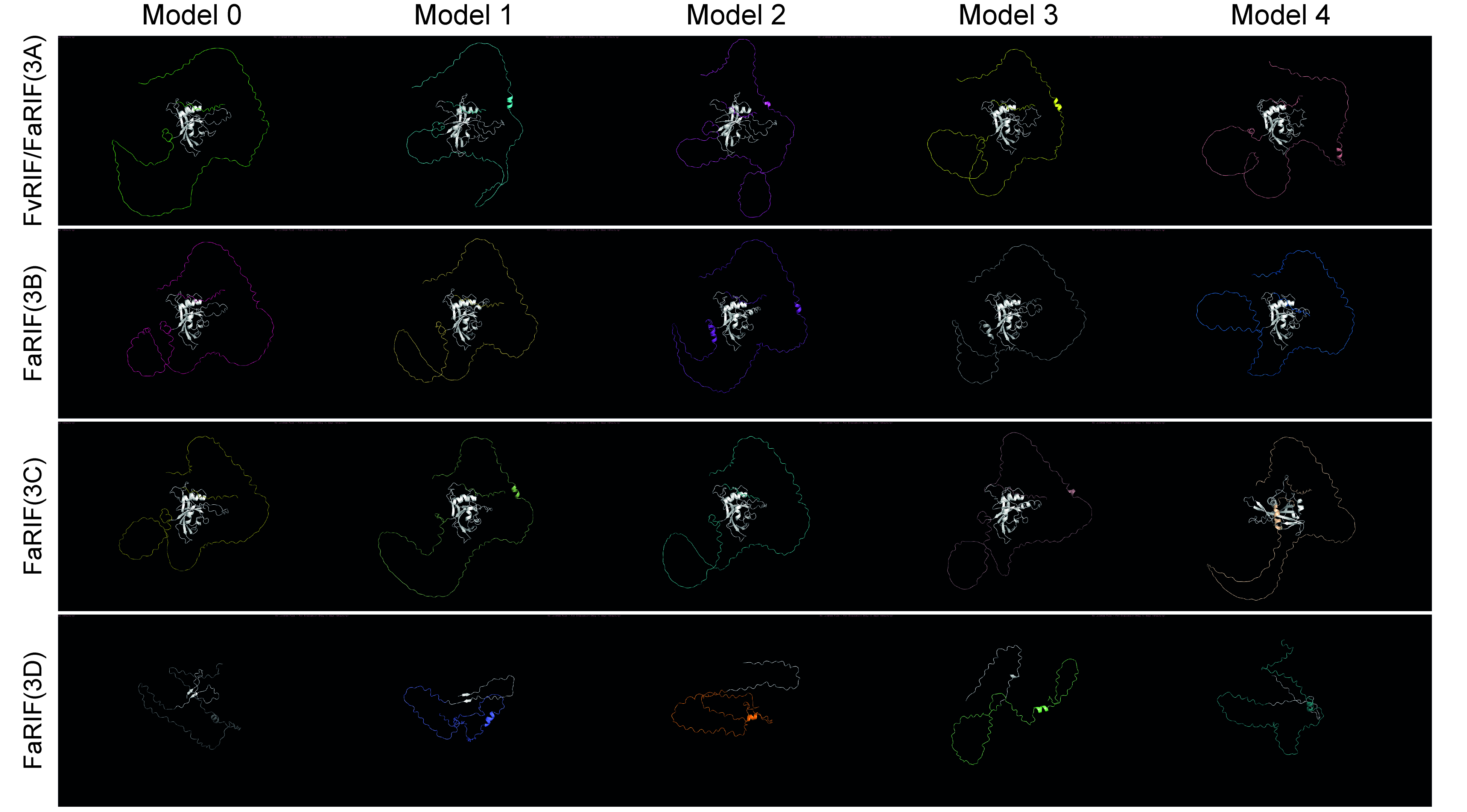

Supplement: Web_Material_uhaf362 [file web_material_uhaf362.zip › Supplementary Fig. S2.tif]

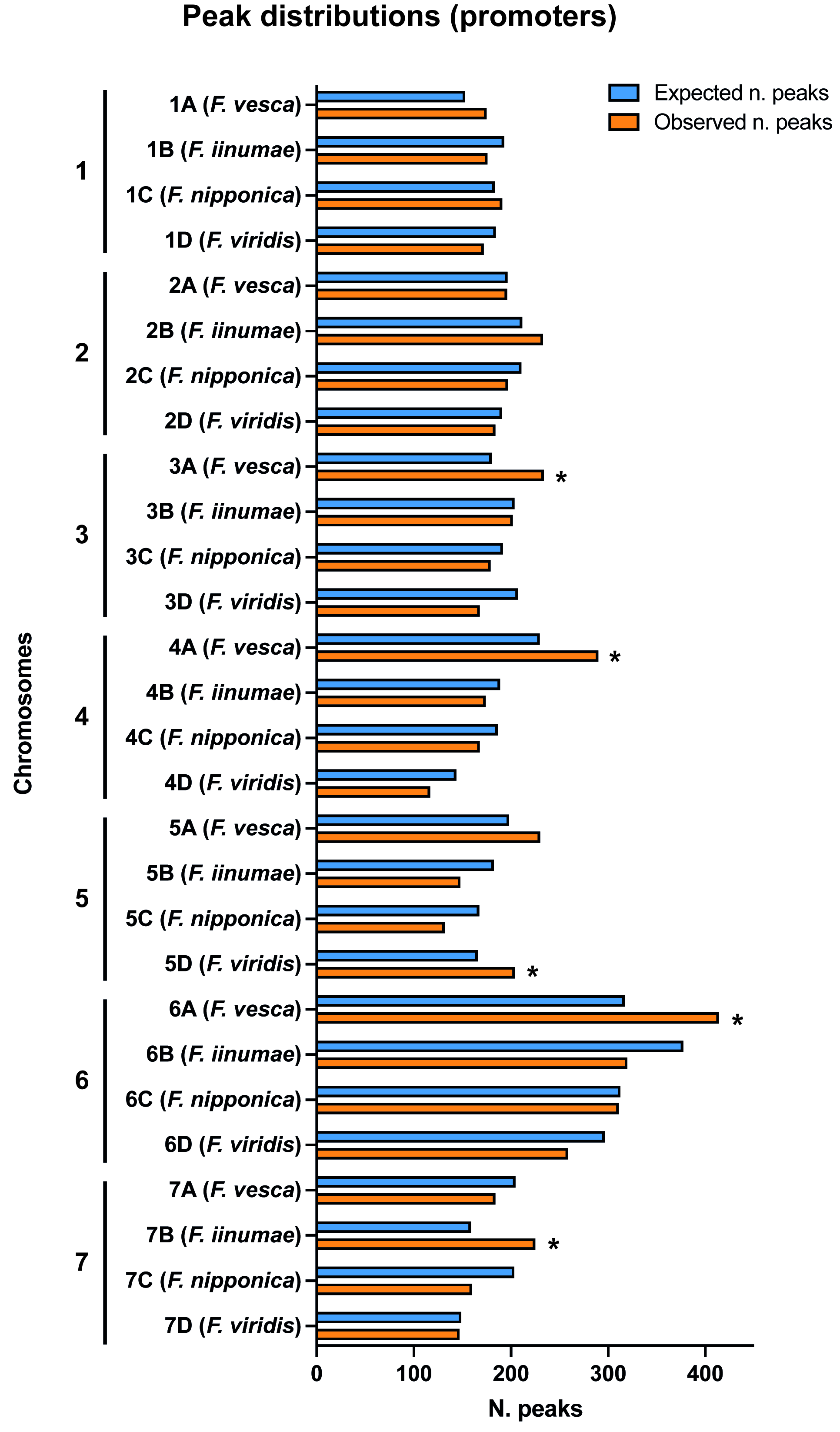

Supplement: Web_Material_uhaf362 [file web_material_uhaf362.zip › Supplementary Fig. S3.tif]

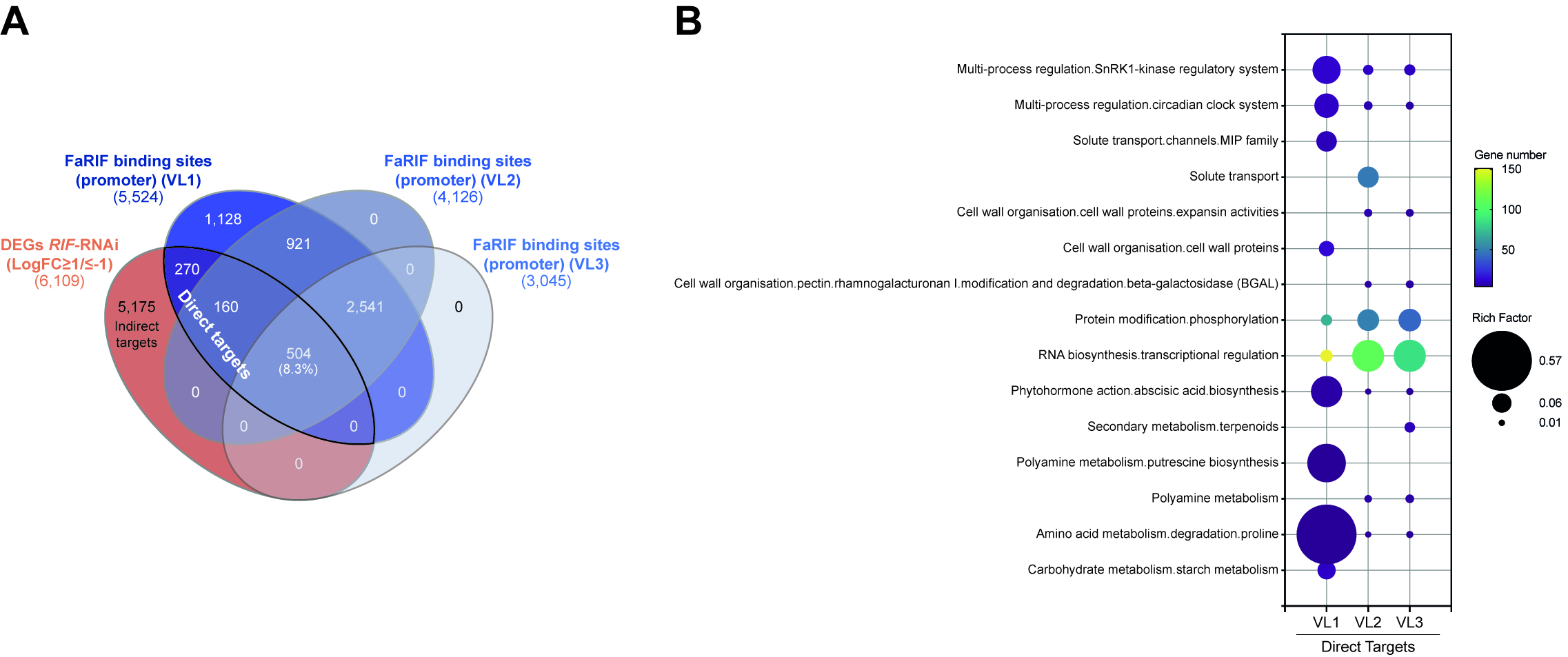

Supplement: Web_Material_uhaf362 [file web_material_uhaf362.zip › Supplementary Fig. S4.tif]

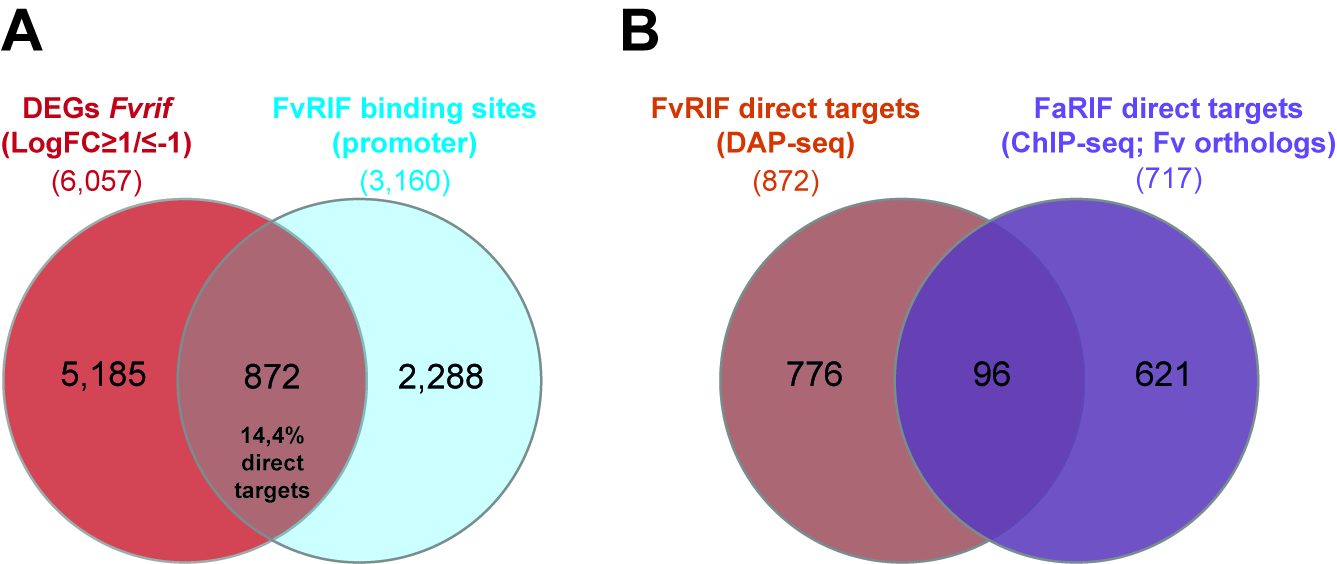

Supplement: Web_Material_uhaf362 [file web_material_uhaf362.zip › Supplementary Fig. S5.tif]

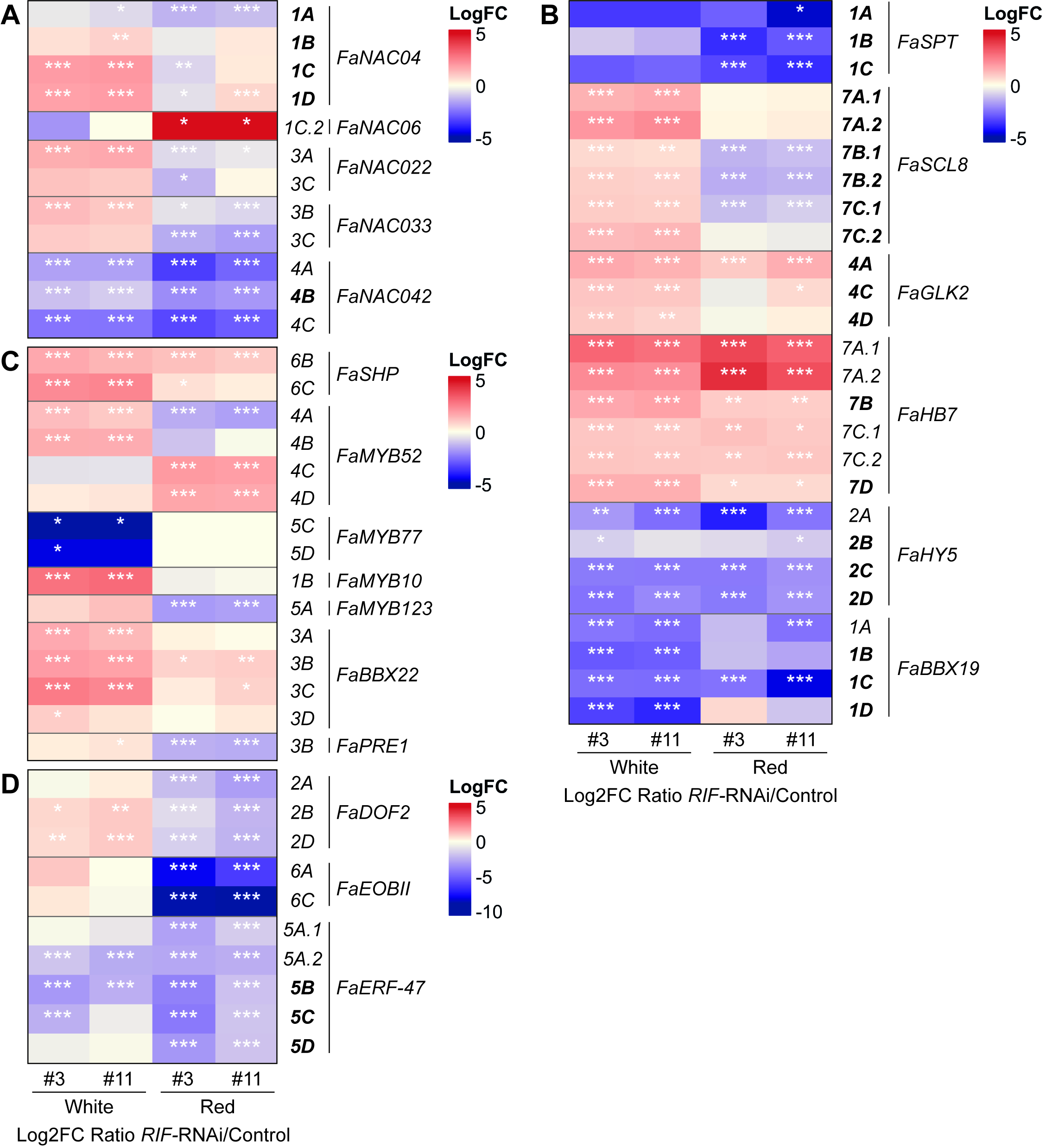

Supplement: Web_Material_uhaf362 [file web_material_uhaf362.zip › Supplementary Fig. S6.tif]

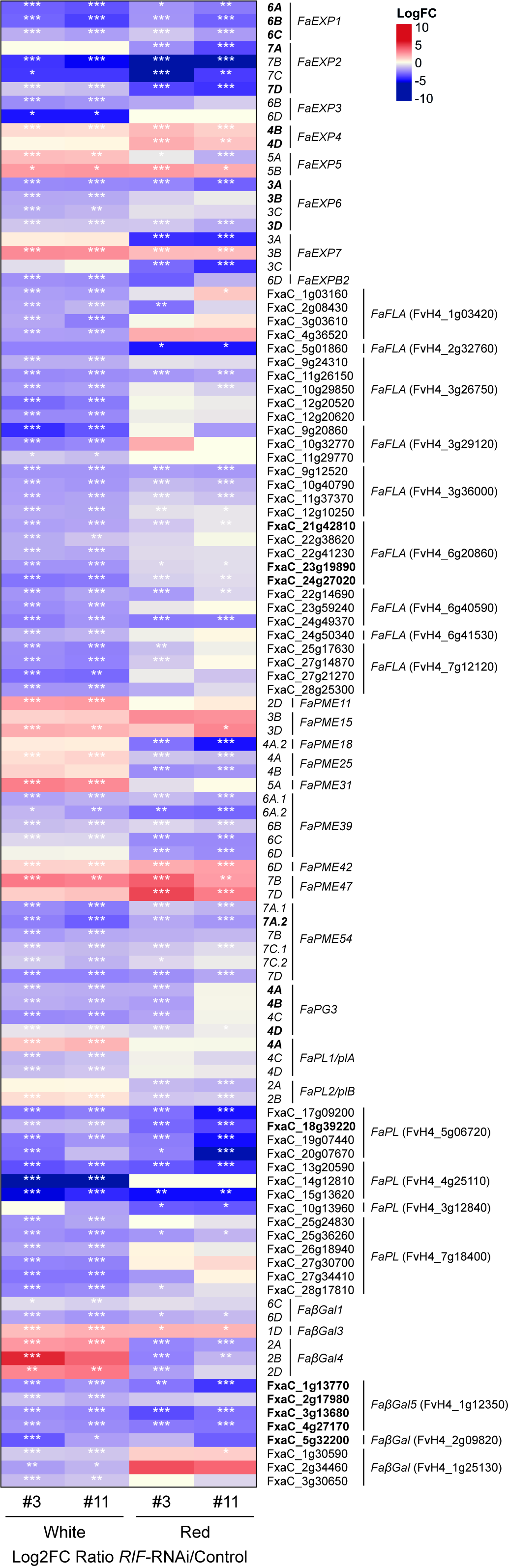

Supplement: Web_Material_uhaf362 [file web_material_uhaf362.zip › Supplementary Fig. S7.tif]

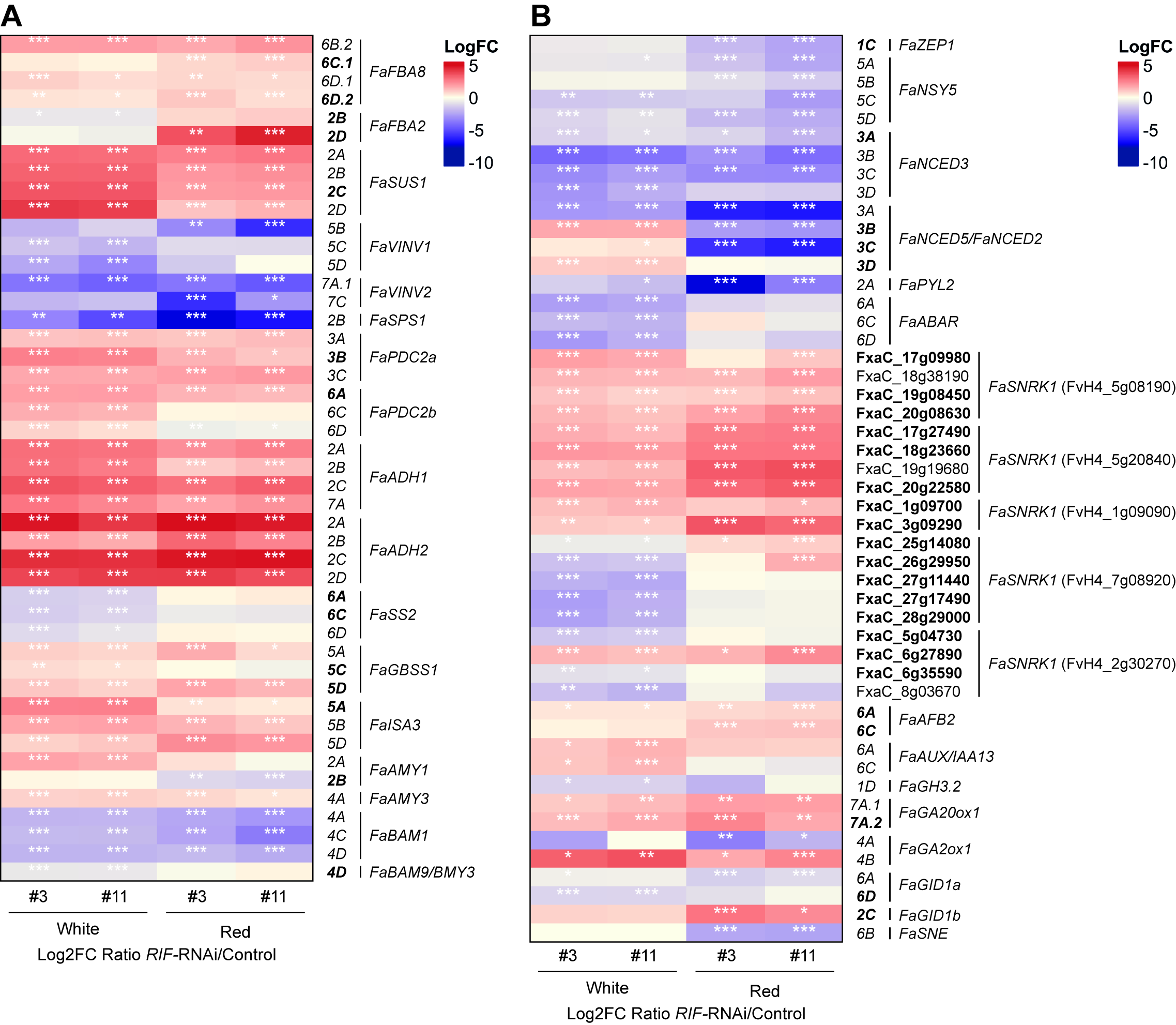

Supplement: Web_Material_uhaf362 [file web_material_uhaf362.zip › Supplementary Fig. S8.tif]

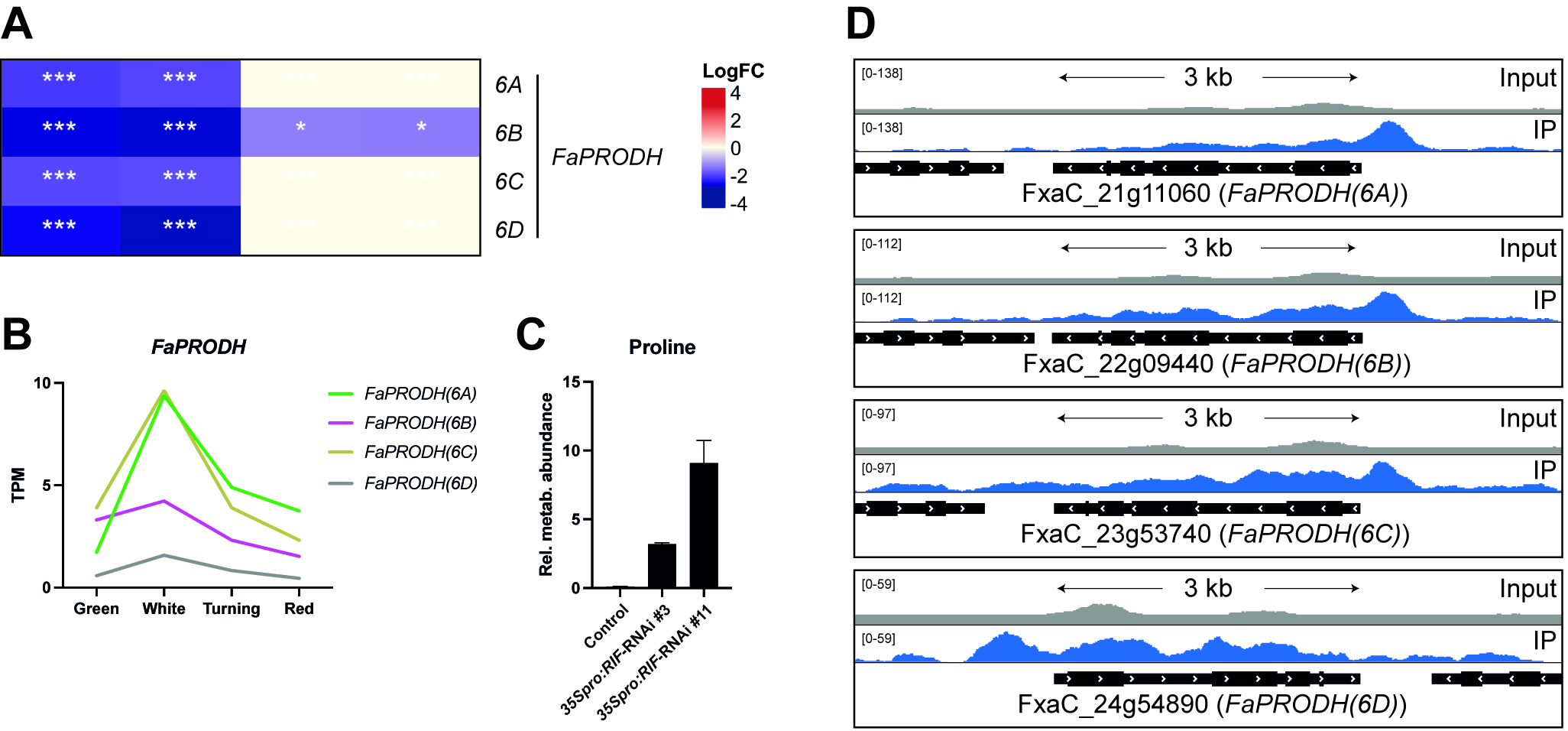

Supplement: Web_Material_uhaf362 [file web_material_uhaf362.zip › Supplementary Fig. S9.tif]

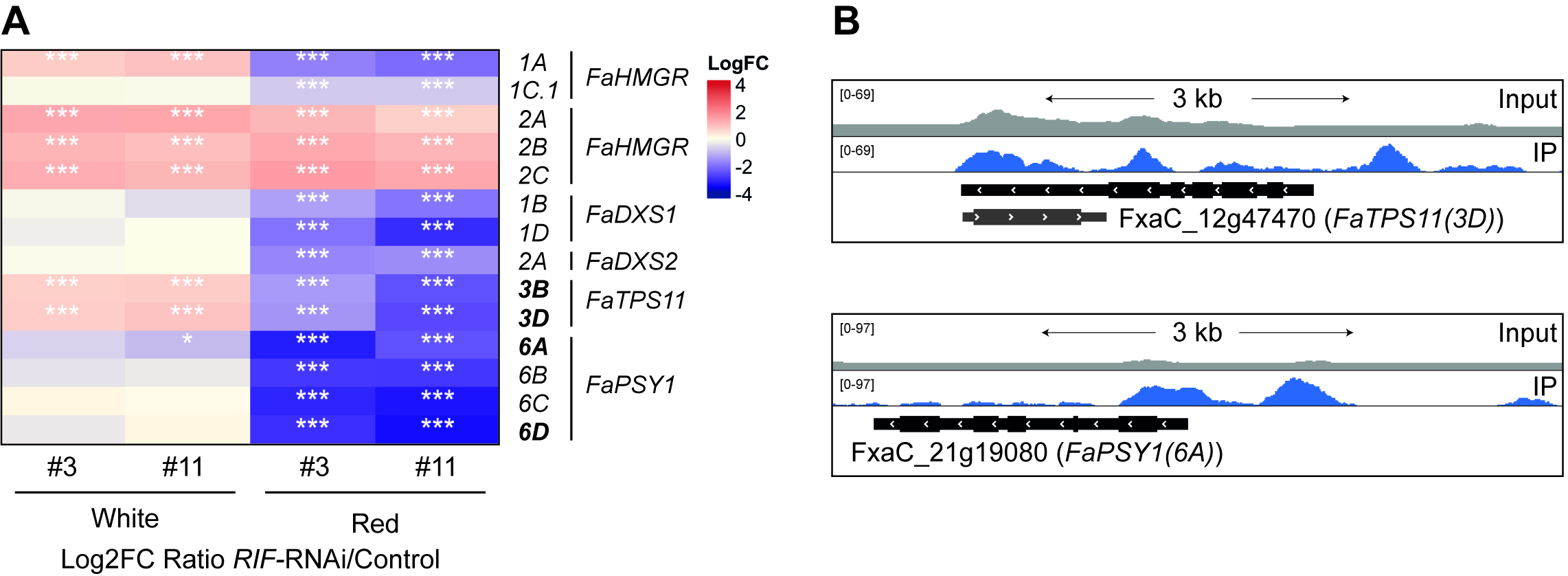

Supplement: Web_Material_uhaf362 [file web_material_uhaf362.zip › Supplementary Fig. S10.tif]

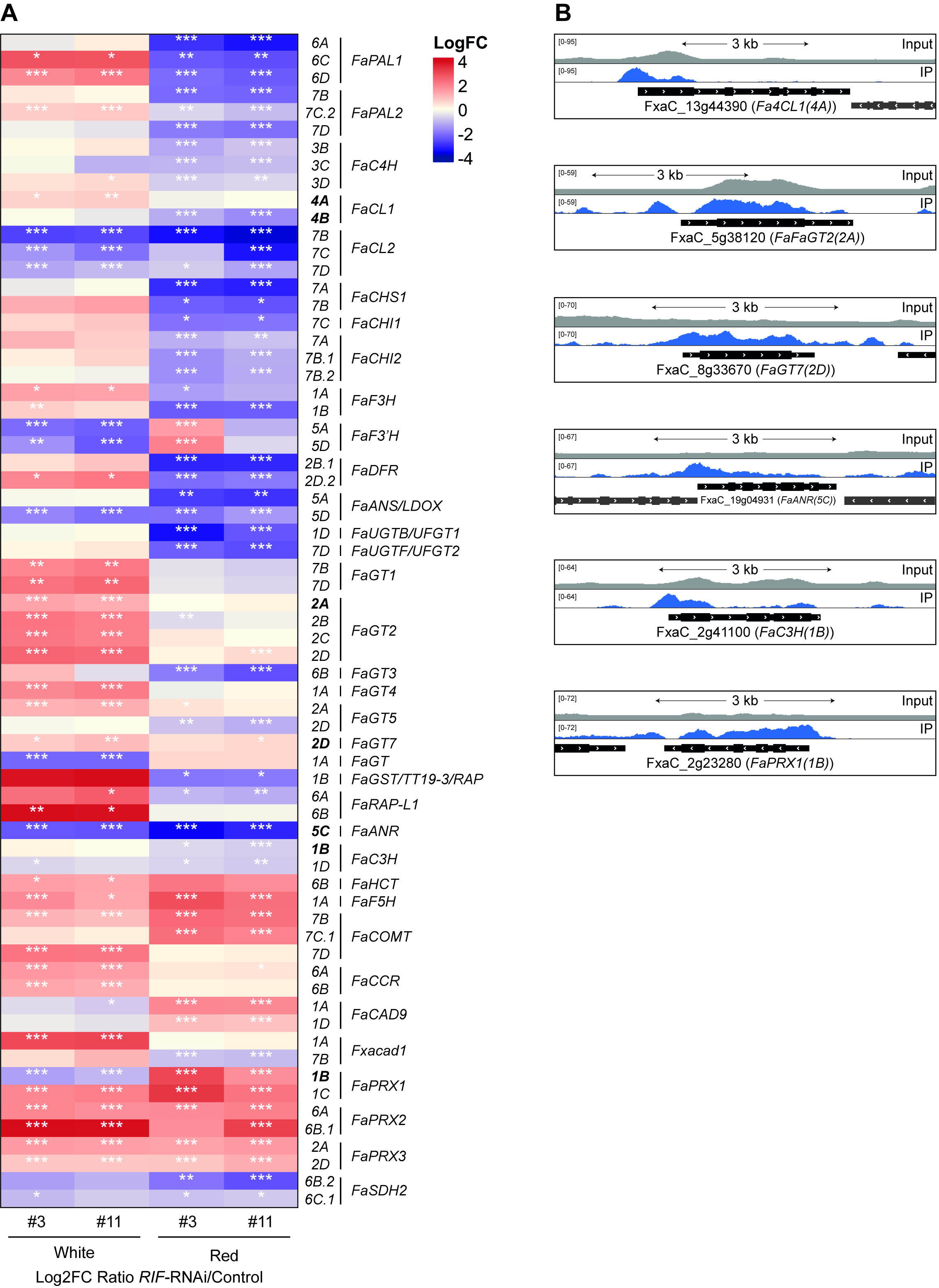

Supplement: Web_Material_uhaf362 [file web_material_uhaf362.zip › Supplementary Fig. S11.tif]

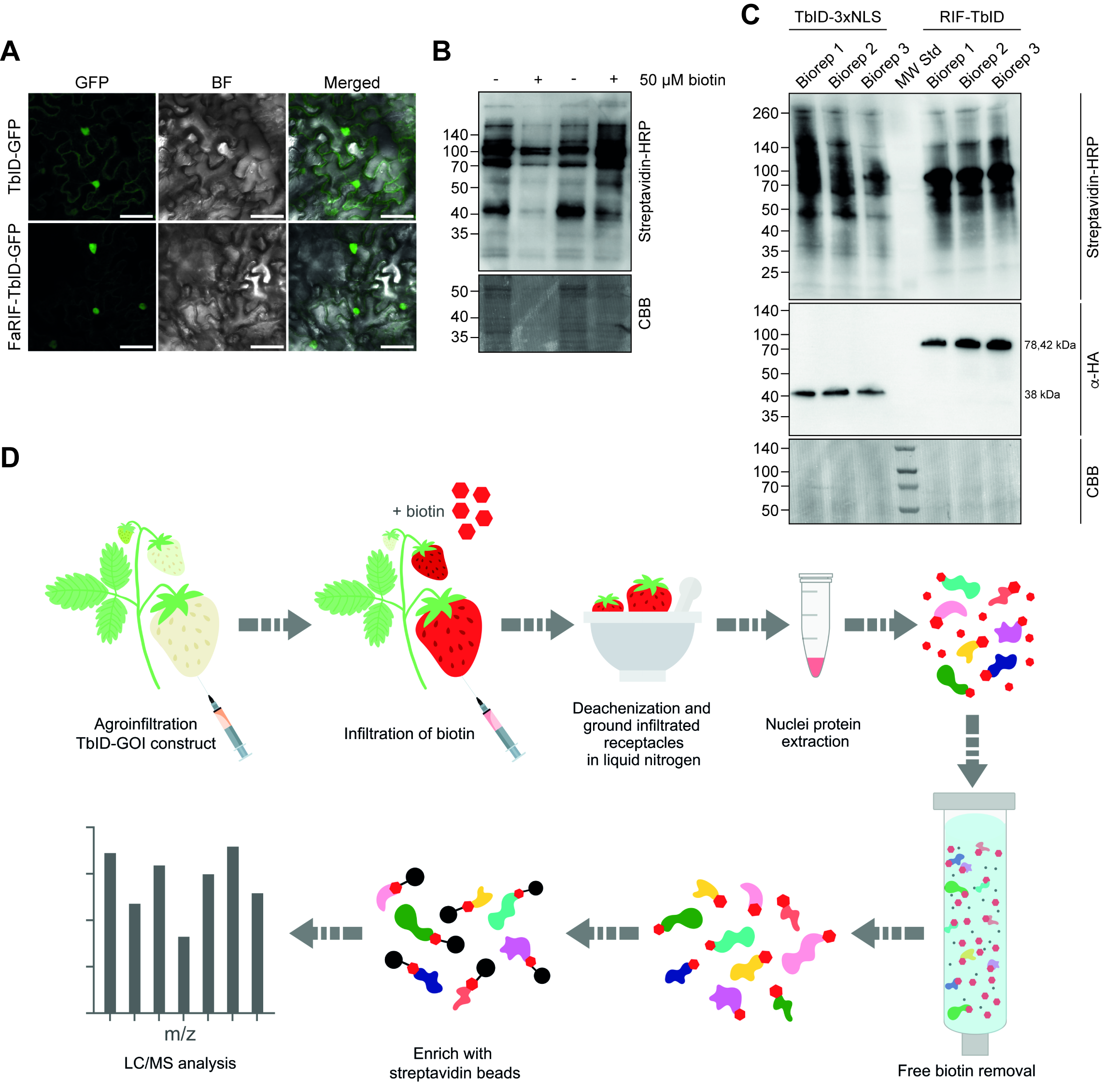

Supplement: Web_Material_uhaf362 [file web_material_uhaf362.zip › Supplementary Fig. S12.tif]
